# ProbStab: A probabilistic ML-assisted pipeline for genotype performance, stability, and risk evaluation in multi-environment trials

**DOI:** 10.1371/journal.pone.0352098

**Published:** 2026-07-10

**Authors:** Mohammadreza Shiri

**Affiliations:** Department of Maize and Forage Crops Research, Seed and Plant Improvement Institute, Agricultural Research Education and Extension Organization (AREEO), Karaj, Iran; KGUT: Graduate University of Advanced Technology, IRAN, ISLAMIC REPUBLIC OF

## Abstract

In multi-environment trials (MET), ignoring genotype × environment interaction (GEI) can lead to suboptimal cultivar recommendations and increased risk in breeding decisions. To make informed decisions, it is important to select genotypes that combine high yield with a low chance of very poor performance. Recent advances in artificial intelligence and machine learning (ML) enable researchers to study complex patterns in MET data, improving the screening and selection of superior genotypes. In this study, we introduce the statistical package ProbStab, which was developed to evaluate the yield, stability, and risk of genotypes in MET. ProbStab is based on Quantile Regression Forests, a non-parametric algorithm that provides reliable prediction intervals and addresses the limitations of classical methods. The package includes several probabilistic modules (ml_tcp_pipe, prob_stab_pipe, ci_perf_pipe, risk_potential_pipe). Using bootstrapping and machine learning, these modules estimate key indicators, including the probability of superior yield, the probability of superior stability, the joint probability of yield and stability, and measures of risk and performance potential. This integrated design fills a crucial gap in conventional approaches, providing a practical tool for advanced probabilistic analysis. By combining MLwith probabilistic evaluation, ProbStab gives plant breeders and agronomists a novel framework to reduce uncertainty in decision-making. The main goal of ProbStab is to support breeding programs with a clear, data-driven, and efficient approach for selecting stable and high-yielding genotypes under complex GEI conditions and to improve the reliability of cultivar recommendations for farmers.

## Introduction

Genotype × environment interaction (GEI) is one of the major challenges in plant breeding programs, as it affects the consistency of genotype performance across environments and complicates the selection of stable, high-yielding cultivars [1–4]. Identifying genotypes that combine both high yield and stability is essential for the commercial success of released varieties. Given the increasing impact of climate change and the global demand for food security, improving the accuracy and reliability of cultivar recommendation has become more critical than ever [3,5].

In practice, the identification and release of superior cultivars is a time-consuming and costly process, typically carried out through multi-stage evaluations that include preliminary yield trials (PYT), advanced yield trials (AYT), and final/VCU (Value for Cultivation and Use) trials. At each stage, data are collected on yield, quality, resistance to pests and diseases, and environmental adaptability [6]. Despite their value, the complexity of genetic and environmental interactions, combined with the large volume of data generated, makes accurate prediction of genotype performance and stability particularly challenging [3]. Most breeding programs continue to rely largely on mean performance, for example, selecting the cultivar with the highest yield estimated by statistical models, and in some cases, complementing this with stability or adaptability analyses [5,7]. However, this dominance of mean-based selection often overlooks the uncertainty caused by environmental variation, which is one of the largest contributors to phenotypic variability in crops.

The concept of incorporating probabilistic approaches to account for GEI dates back several decades [8–10]. Early studies demonstrated the potential of using probabilistic ideas to analyze GEI [11–13], but their practical application in plant breeding has remained limited. A probabilistic alternative for cultivar selection is to view environments as random and to choose cultivars that are most likely to perform well across a set of target environments, rather than simply selecting the cultivar with the highest mean. From a decision-making perspective, this is more consistent with risk-averse strategies, where breeders aim to maximize the probability of success under uncertainty [11–13]. For instance, a cultivar that performs extremely well in a few favorable environments may have a high mean yield, but another cultivar with more consistent performance may have a higher probability of success across a wider range of environments. Importantly, Tohidi and Olafsson [14] demonstrated that no single existing stability measure can fully capture these differences between mean and probabilistic ranks, highlighting the need for integrated probabilistic and non-parametric approaches, such as Quantile Regression Forests (QRFs) combined with bootstrap resampling. Probabilistic ranking of cultivars can effectively combine both mean performance and stability across environments, providing a more robust basis for selection [14]. This approach considers the probability that a genotype will outperform others in a random set of target environments, thereby directly incorporating environmental uncertainty into decision-making. Notably, the study reveals that the differences between mean-based and probabilistic rankings are largely attributed to variability in genotype performance, highlighting that a genotype with a high mean yield in favorable environments may not always be the most reliable choice.

To operationalize this idea, probabilistic analyses and risk quantification become essential. Bootstrap resampling, for example, is an effective statistical approach that generates thousands of resampled datasets from observed data [15,16], allowing for the estimation of probability distributions for performance and stability indices [14,17–19]. This enables the quantification of both the probability of genotype superiority and the associated selection risk, thereby supporting evidence-based decisions rather than relying on point estimates [20,21]. Earlier studies [22,23] and more recent bootstrap-based approaches [18] have demonstrated the potential of probabilistic comparisons; however, they still fall short in integrating multi-stage data and achieving high predictive accuracy.

Bayesian methods, such as those implemented in ProbBreed, represent another class of probabilistic approaches in which prior information is combined with observed data to generate posterior distributions [21,24–27]. While flexible, their performance can be sensitive to prior specification, particularly when strong informative priors are used. However, Bayesian models can also employ uninformative or objective priors, allowing the data to dominate the posterior distribution. Poor prior selection remains a concern primarily when subjective or poorly calibrated informative priors are applied [21,24]. Furthermore, Bayesian methods such as ProbBreed are often demonstrated using final/VCU trials data [25]. While these methods have the ability to integrate earlier stage data through the inclusion of year effects, in practice, the sets of genotypes evaluated in PYT, AYT, and final/VCU trials are not identical. Many genotypes are discarded after each stage, and experimental designs often differ. Consequently, integrating non-identical genotype sets across multiple stages with different structures is not straightforward and requires careful manual effort. As a result, these models often struggle to discern complex patterns, which diminishes their predictive accuracy. That is a major reason why many breeding programs still rely primarily on final trial data. ProbStab is explicitly designed to overcome this limitation by using QRF, which can learn from partially overlapping genotype sets across all three stages without requiring balanced or identical structures.

Recent advances in machine learning (ML) and artificial intelligence provide new opportunities to address these challenges. Among these, QRFs have gained attention due to their non-parametric nature, ability to capture non-linear relationships, and provision of prediction intervals [28]. A key strategy in this context is the integrated use of data from all three evaluations – PYT, AYT, and final/VCU trials – as input for ML models. Leveraging the comprehensive three-stage datasets enhances the accuracy of final performance predictions and supports more informed breeding decisions. However, to our knowledge, no studies have explicitly explored the combined use of multi-stage trial data – PYT, AYT, and final/VCU trials – with non-Bayesian probabilistic ML approaches such as Quantile Regression Forests. Previous ML applications have focused on prediction or classification tasks [15,19], leaving a significant research gap for probabilistic integration of multi-stage data.

The approach proposed in this study, built on a non-Bayesian framework and QRF, addresses these limitations. It utilizes three-stage data (PYT, AYT, and final/VCU trials) to enhance genetic and environmental diversity in the training dataset. Training the model on all available data improves prediction accuracy. Additionally, bootstrap methods facilitate the quantitative estimation of uncertainty and recommendation risk. These innovations are implemented in the ProbStab software package, a clear, accurate, and reproducible tool that, while retaining some of the strengths of Bayesian approaches, leverages all three-stage data and the non-parametric nature of QRF to significantly improve predictive accuracy and reliability. Therefore, the objectives of this study were to: (i) integrate multi-stage yield trial data into a QRF framework, (ii) quantify prediction uncertainty and selection risk through bootstrap resampling, and (iii) implement these innovations in an open-source software (ProbStab) to support evidence-based breeding decisions.

## Methods

### Theoretical basis for multi-environment trial data analysis using the ProbStab framework

In MET, the primary goal is to select genotypes that exhibit high yield and stable performance across diverse environments, while also accounting for uncertainty and risk in the selection process. Choosing and recommending genotypes for target environments always comes with hidden risks that plant breeders face [29].

In the ProbStab software package, uncertainty is quantified using bootstrap resampling. This method creates thousands of resampled datasets (equal to the number of bootstrap repetitions) to estimate the probability distributions of performance and stability indices for each genotype. Using this approach, breeders can quantify both the probability of genotype superiority and the associated selection risk, enabling decisions to be based on probabilistic evidence and stability, rather than relying on point estimates.

**Fig 1:**
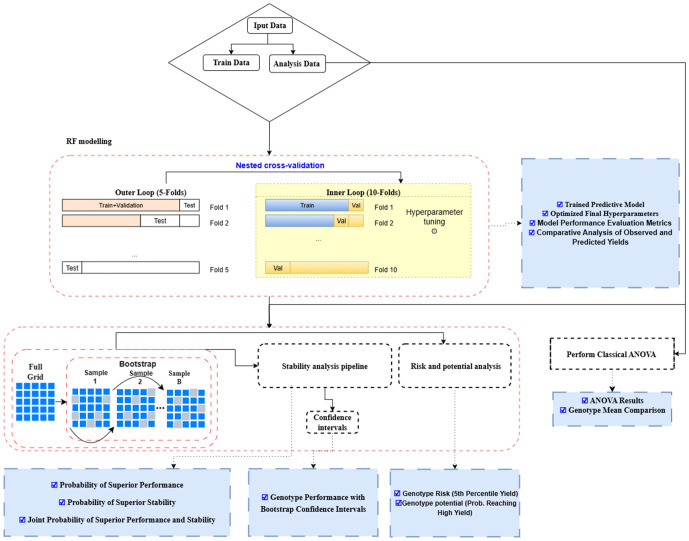
Overview of the ProbStab framework for probabilistic assessment of genotype performance and stability.

The non-parametric nature of QRF in ProbStab enhances the accuracy and reliability of genotype recommendations. To address the complexity inherent in GEI within MET, ProbStab integrates ML with stability analysis. The workflow encompasses the following steps: (1) Random Forest modeling of complex GEIs to predict genotype performance, and (2) Bootstrap resampling to estimate empirical probabilities of superior performance and/or stability of genotypes in the resampled datasets. This integrated approach enables the simultaneous evaluation of genotype performance and stability, providing a strong selection criterion for breeding programs (Fig 1).

### Random forest modeling and performance prediction

To predict genotype performance in different environments, an **RF** model was used. This model builds multiple decision trees on bootstrap samples and random subsets of predictors and averages the predictions from all trees, which increases accuracy and reduces overfitting.

The predicted performance of genotype *i* in environment *j* is defined as [28]:


$$\begin{array}{c} {\hat{\text{Y}}}_{ijk}=f(Genotype\_i,Environment\_j)+\varepsilon_{ijk} \end{array}$$

where *f*(⋅) is the non-parametric function learned by the RF, and $\varepsilon_{ijk}$ is the residual error.

This algorithm represents an advanced extension of the RF, estimating the complete distribution of responses (in this context, performance) rather than solely the mean. Consequently, it facilitates the calculation of quantiles and the evaluation of predictive uncertainty, rendering QRF particularly suitable for analyzing variable or complex datasets.

### Hyperparameter optimization and model calibration

Hyperparameter optimization was conducted using an exhaustive grid search combined with k-fold cross-validation (CV) to ensure robust parameter selection and to mitigate overfitting. Specifically, the training dataset was partitioned into *k* folds (for example, k = 5), where in each iteration, four folds were used to train the model and the remaining fold was used for validation. This process was repeated across all folds, such that each fold served as a validation set once.

For each candidate hyperparameter configuration in the search grid, comprising the number of randomly sampled predictors at each split (mtry) and the minimum node size (min.node.size), the model’s predictive performance was assessed via cross-validation. The configuration yielding the lowest prediction error and highest coefficient of determination (R²) was selected as the optimal set of hyperparameters. The split rule was fixed to “variance,” and bootstrap resampling was employed with a sampling fraction of 0.632, consistent with standard RF methodology [30].

It is generally recommended to employ a sufficiently large number of trees in the forest to stabilize predictions and reduce variance. The default value in ProbStab is 1000 trees, which balances predictive accuracy and computational efficiency. Users may adjust this parameter according to their data size and available computational resources [31–33].

Following hyperparameter selection, the final RF model was refitted on the full training dataset using the optimal parameter configuration. Predictive performance was then evaluated on the independent analysis dataset.

### Evaluation of random forest model prediction performance

To evaluate the prediction performance of the RF model for estimating genotype performance, three common statistical metrics were used: Mean Error (ME), Root Mean Square Error (RMSE), and Coefficient of Determination (R²).

In addition to these numerical metrics, a density plot of predicted versus observed values was constructed to visually assess the agreement between predicted and real observations in the test data. These metrics were calculated for each fold and reported as an average to indicate the model’s generalization accuracy.

### Quantifying prediction uncertainty of the model

To evaluate the uncertainty associated with RF predictions, QRFs were employed [34]. QRF estimates the conditional distribution of the response variable and allows the construction of prediction intervals (PIs) based on specific quantiles of this distribution.

To quantify prediction uncertainty, the 0.05 and 0.95 quantiles of the conditional distribution were used. The prediction interval width (PIW) is defined as: PIW = q0.95 − q0.05, where q0.95 and q0.05 represent the 95th and 5th percentiles, respectively. The PIW indicates the range of uncertainty around each prediction. These quantiles correspond to a 90% prediction interval, a standard level in ML uncertainty quantification that balances interval width and coverage probability [35].

To assess the accuracy of these uncertainty estimates, the Prediction Interval Coverage Probability (PICP) was calculated for different interval levels. PICP is the proportion of observed values that fall within the corresponding prediction interval. For example, for PI90, a well-calibrated model should have a PICP close to 0.90, meaning that approximately 90% of observed values lie within the interval [36].

Significant deviations between PICP and the nominal PI level indicate miscalibration: if PICP is lower than nominal, the model underestimates uncertainty, while if PICP is higher than nominal, the model overestimates uncertainty [16].

In addition to PICP, the Mean Prediction Interval Width (MPIW) is commonly utilized as a performance metric in statistical and ML models. MPIW reflects the model’s capacity to minimize prediction uncertainty and is calculated as follows [35]:


$$\begin{array}{c} MPIW=\frac{\text{1}}{n}\sum_{i=\text{1}}^{n}q\text{0}.\text{9}\text{5}(x_{i})-q\text{0}.\text{0}\text{5}(x_{i}) \end{array}$$

where *n* is the number of observations, and *q*0.95(*x_i_*) and *q*0.55(*x_i_*) are the 95th and 5th percentiles of the conditional distribution of the response trait for observation *i*.

The acceptable range for MPIW depends on the context and type of prediction. Generally, a smaller MPIW indicates more precise predictions, while a larger MPIW reflects greater uncertainty. As a guideline, if the MPIW is less than 10% of the observed range of the trait, it is considered good; values between 20–30% are acceptable, and values above 40% indicate that prediction intervals are excessively wide [37].

### Probability of superior performance

Let *B* denote the total number of bootstrap iterations and *G* the complete set of genotypes evaluated. In each iteration, b ∈ {1, 2,  ..., B}, a dataset is generated by resampling the original data, and a prediction model is fitted. Based on model predictions, a subset T_b_ of top-performing genotypes is identified according to the specified selection intensity (e.g., 15%) based on predicted performance.

To determine whether a specific genotype *g* belongs to T_b_, the following indicator function is used:


$$\begin{array}{c} I\left(g\in T_{b}\right)=\left\{ \begin{array}{c} \text{1},ifg\in T_{b}\\ \text{0},otherwise \end{array}\right. \end{array}$$

The probability of superior performance for genotype g is calculated as:


$$\begin{array}{c} {\text{P}}_{\text{g}(\text{p})}=\frac{\text{1}}{\text{B}}\sum_{b=\text{1}}^{B}I\left(g\in T_{b}\right) \end{array}$$

This metric represents the relative frequency with which genotype *g* appears among the top genotypes across all bootstrap iterations, thereby providing a non-parametric, robust, and reliable estimate of performance that accounts for sampling uncertainty and model variability. Higher P_g(p)_ values indicate superior predicted performance across diverse environments.

### Probability of superior stability

A stable genotype is traditionally defined as one exhibiting lower variance in GE, which can be estimated using Shukla’s stability variance for each genotype. Shukla’s stability variance (σ²ᵢ), also known as the stability variance statistic, estimates the contribution of each genotype to the overall genotype × environment interaction. It is calculated as the squared deviation of each genotype’s performance across environments, adjusted for the interaction mean square. This parameter was chosen because it is computationally straightforward, widely used in plant breeding literature, and provides a genotype-specific stability measure that is independent of the overall mean [25]. In each bootstrap iteration, stable genotypes are those with the lowest stability variance values, forming a subset S_b_ of the genotypes selected at the specified selection intensity (e.g., 15%).

The indicator function for determining whether genotype *g* is among the most stable genotypes (S_b_) is:


$$\begin{array}{c} I\left(g\in S_{b}\right)=\left\{ \begin{array}{c} \text{1},ifg\in S_{b}\\ \text{0},otherwise \end{array}\right. \end{array}$$

The probability of superior stability for genotype *g* is then calculated as:


$$\begin{array}{c} {\text{P}}_{\text{g}(\text{S})}=\frac{\text{1}}{\text{B}}\sum_{b=\text{1}}^{B}I\left(g\in S_{b}\right) \end{array}$$

This probability reflects the frequency with which genotype *g* appears among the most stable genotypes across bootstrap samples, thereby providing a robust estimate of stability that accounts for both sampling and model prediction uncertainty. It is particularly valuable for identifying genotypes exhibiting consistent performance under variable or unpredictable environmental conditions.

### Joint probability of performance and stability

To estimate the probability that a genotype is simultaneously among the highest-performing and most stable genotypes, in each iteration, the subsets T_b_ and S_b_ represent the top-performing and most stable genotypes at the defined selection intensity. Their intersection is defined as:


$$\begin{array}{c} J_{b}={\text{T}}_{\text{b}}\cap {\text{S}}_{\text{b}} \end{array}$$

The joint indicator function is


$$\begin{array}{c} I\left(g\in J_{b}\right)=\left\{ \begin{array}{c} @l\text{1},ifg\in J_{b}\\ \text{0},otherwise \end{array}\right. \end{array}$$

The joint probability of superior performance and stability for genotype *g* is:


$$\begin{array}{c} {\text{P}}_{\text{g}(\text{j})}=\frac{\text{1}}{\text{B}}\sum_{b=\text{1}}^{B}I\left(g\in J_{b}\right) \end{array}$$

This metric captures the simultaneous achievement of high performance and stability for genotype *g* across all bootstrap iterations, serving as a comprehensive index for selecting candidate genotypes best suited for breeding programs that target stable performance in variable environments.

### Risk and potential of genotypes

Let B_CI_ denote the number of bootstrap iterations for evaluating genotype risk and potential. In each iteration, b ∈ {1, 2,  ..., B_CI_}, a resampled dataset is generated, and the prediction model is fitted. Predicted genotype performance across environments is then computed.

Genotype “risk” is defined as an indicator of low performance. For each genotype *g*, in each bootstrap iteration, the lower *5th* percentile of predicted performance is calculated:


$$\begin{array}{c} \overset{\left(b\right)}{\underset{g,\text{5}\%}{\hat{Y}}}=Q_{\text{0}.\text{0}\text{5}}(\overset{\left(b\right)}{\underset{g}{\hat{Y}}}) \end{array}$$

where $\overset{\left(b\right)}{\underset{g,\text{5}\%}{\hat{Y}}}=Q_{\text{0}.\text{0}\text{5}}(\overset{\left(b\right)}{\underset{g}{\hat{Y}}})$ represents predicted performance in all environments for iteration *b*, and *Q0.05* denotes the *5th* percentile. Genotype risk is calculated as the average across all iterations:


$$\begin{array}{c} {Risk}_{g}=\frac{\text{1}}{B_{CI}}\sum_{b=\text{1}}^{B_{CI}}\overset{\left(b\right)}{\underset{g,\text{5}\%}{\hat{Y}}} \end{array}$$

A lower ${Risk}_{g}$ indicates greater sensitivity to environmental conditions and a higher likelihood of poor performance.

Genotype “potential” is defined as an indicator of high-performance capacity. For each genotype *g,* in each bootstrap iteration, the upper *80th* percentile of predicted performance is computed:


$$\begin{array}{c} \overset{\left(b\right)}{\underset{g,\text{8}\text{0}\%}{\hat{Y}}}=Q_{\text{0}.\text{8}\text{0}}(\overset{\left(b\right)}{\underset{g}{\hat{Y}}}) \end{array}$$

The genotype potential is the mean across all bootstrap iterations:


$$\begin{array}{c} {Potential}_{g}=\frac{\text{1}}{B_{CI}}\sum_{b=\text{1}}^{B_{CI}}\overset{\left(b\right)}{\underset{g,\text{8}\text{0}\%}{\hat{Y}}} \end{array}$$

Higher ${Potential}_{g}$values indicate greater performance capacity.

The 5th percentile was chosen to represent the ‘worst-case scenario’ or lower bound of expected performance, which is consistent with risk-averse selection strategies in plant breeding [22]. The 80th percentile was chosen to represent high-performance potential while avoiding the instability of extreme upper quantiles (e.g., 95th percentile), which may be overly sensitive to outliers.

For each genotype, the probability of exceeding the *80th* percentile threshold -based on the mean *80th* percentile across all genotypes- is calculated:


$$\begin{array}{c} P_{high(g)}=\frac{\text{1}}{B_{CI}}\sum_{b=\text{1}}^{B_{CI}}{I(Trsehold}_{\text{8}\text{0}\%}\leq \overset{\left(b\right)}{\underset{g,\text{8}\text{0}\%}{\hat{Y}}}) \end{array}$$

The indicator function *I* (⋅) is:


$$\begin{array}{c} I(condition)=\left\{ \begin{array}{c} @c\text{1},iftheconditionissatisfied\\ \text{0},otherwise \end{array}\right. \end{array}$$

This metric reflects the frequency with which genotype *g* surpasses the defined threshold, thereby assisting in the selection of genotypes with high, stable performance while accounting for sampling uncertainty and model variability.

### Confidence interval estimation

The 95% confidence intervals (CIs) for the predicted performance of each genotype were estimated using a bootstrap approach. Let B_CI_ denote the number of bootstrap iterations. In each iteration, a resampled dataset is generated from the data with replacement, and a RF model is refitted using the optimized hyperparameters. The predicted performance for each genotype across all environments is calculated, and the mean predicted yield is recorded. After all iterations, the *2.5th* and *97.5th* percentiles of the predicted yields across bootstrap samples are used as the lower and upper bounds of the 95% CI, respectively, while the mean provides the average predicted performance. Genotypes are classified as “Above Average” or “Below Average” depending on whether their mean predicted yield exceeds or falls below the grand mean across genotypes.

This approach accounts for sampling variability and model uncertainty, thereby providing a robust estimate of prediction accuracy. The results can be visualized in a plot depicting the mean predicted yield, confidence intervals, and the grand mean reference, thereby facilitating the comparison of genotypes across multiple environments.

For comparing genotypes with each other or with a check, the confidence intervals offer a straightforward visual criterion: if the confidence intervals of two genotypes (or a genotype and a check) overlap, the difference is not statistically significant at the 95% confidence level.

### Software implementation

The stable version of the ProbStab package will be available from CRAN (https://CRAN.R-project.org/package=ProbStab) and can be installed directly via the R console using the following command:

install.packages(“ProbStab”)

The development version of the package will also be available on GitHub (https://github.com/Mohammadreza-shiri/ProbStab) and can be installed with the devtools package:

# install.packages(“devtools”) # Run this line if devtools is not installed

devtools::install_github(“Mohammadreza-shiri/ProbStab”)

After installation, the package may be loaded using:

library(ProbStab)

### Motivational example

The following example demonstrates the application of the ProbStab package. The dataset used in this example comes from a maize breeding program conducted in Iran. The final/VCU trial included 11 maize test hybrids evaluated across 20 environments (location-year combinations) using a Randomized Complete Block Design (RCBD) with four replications. The preliminary trials comprised 99 hybrids evaluated using an alpha lattice design with two replications at three locations, and the intermediate trials comprised 19 hybrids evaluated using an RCBD with three replications at four locations.

To load and prepare the data, two datasets are required. The first, the training dataset, contains all experimental data, including PYT, AYT, and final yield/VCU trials. The second, the analysis dataset, comprises only the data from the final/VCU trials. The training dataset, in addition to the analysis dataset, contains data from the preliminary trials and intermediate trials. Notably, the hybrids tested in the final/VCU trial are also included among those evaluated in the PYT and AYT trials.

Columns in the datasets are organized by environment, replication (rep), genotype, and yield, with genotype and environment defined as factors. Raw phenotypic data (plot-level yield) from each environment are used directly for model training and hyperparameter tuning. No prior adjustment (e.g., spatial correction or mixed-model adjustment) is applied to the data before RF modeling. The QRF algorithm learns environmental effects implicitly through the environment predictor variable, which is treated as a categorical factor. This approach maintains transparency and avoids additional parametric assumptions.

The QRF algorithm does not rely on any statistical design assumptions. It learns purely from the predictor variables (environment and genotype) and does not require information about experimental design, blocking, or replication structure. The replication column is included in the dataset because any experimental design (RCBD, lattice, alpha design, etc.) includes replication. Therefore, ProbStab can analyze data from any experimental design without modification. If a user has unreplicated data, they can simply place the constant value 1 in the replication column. The replication column is ignored by the QRF model and is only used for the optional classical ANOVA function (perform_anova), which is provided separately for users who wish to compare ML results with traditional analysis.

To execute the program, the function load_data is called to read and format the training and analysis datasets from the specified paths. This function prepares the data and returns it as a list.

**Box 1. Usage of the function load_data**

met_data < - load_data(

training_path = “Enter the path to the training dataset here,”

analysis_path = “Enter the path to the analysis dataset here”

)

**Note:** If the researcher has access only to the final trial data, both files – the training and analysis datasets – can be regarded as identical.

In addition to the probabilistic ML pipeline, ProbStab also provides a classical ANOVA function (perform_anova) for users who wish to compare the results with traditional stability analysis.

### Comparison ProbStab with ProbBreed

To benchmark ProbStab against an established probabilistic approach, a direct empirical comparison was conducted using the same maize dataset. ProbStab was run with 1000 bootstrap iterations (default setting in prob_stab_pipe, ci_perf_pipe, and risk_potential_pipe). To match this sampling effort, the ProbBreed package [25] was applied to the final/VCU trial data using a Bayesian multi‑environment model (genotype and environment as fixed effects, including genotype × environment interaction). Four MCMC chains were run, each with 500 iterations; the first 50% of iterations (250 samples per chain) were discarded as burn‑in, resulting in a total of 1000 posterior samples for inference. To ensure comparability, a selection intensity of 15% was used in both methods, matching the default setting in ProbStab (top_geno_frac = 0.15). The results obtained from ProbBreed are presented as highest posterior density (HPD) intervals for genotypic main effects (Supplementary S2 Fig), along with the probability of superior performance (Supplementary S3 Fig), the probability of superior stability (Supplementary S4 Fig), and the joint probability of superior performance and stability (Supplementary S5 Fig).

## Results

### Hyperparameter tuning and model evaluation

In the first step of this analysis, after loading and preparing the data using the load_data function and introducing the training and analysis datasets, the QRF model was trained. Hyperparameter tuning via cross validation prevented data leakage, and the default number of trees was set to 1000 (user modifiable) for stable predictions. All these steps were carried out using the ml_tcp_pipe function (Box 1). Depending on the dataset and the breeder’s strategy, the user can change the default values and the input hyperparameter grid as needed (Box 2).

**Box 2. Usage of the function ml_tcp_pipe**

ml_tcp_pipe(

df_train = met_data$train,

df_analysis = met_data$analysis,

mtry_values = c(1, 2),

node_size_values = c(3, 5, 10),

cv_folds = 5,

num_trees = 1000

)

### Optimal hyperparameters of RF for yield prediction

By running the ml_tcp_pipe function, the best model hyperparameters were obtained. These can be viewed with the command: print(analysis_results$hyperparameters); and are summarized in Table 1.

**Table 1:** Final Hyperparameters Used for the Model.

Hyperparameter	Value
**mtry**	**2**
**Node size (minimum.node.size)**	**5**
**Replace**	**True**
**Split Rule**	**variance**
**sample.fraction**	**0.63**

Another output of the ml_tcp_pipe function is the evaluation of the model performance for both the training and analysis datasets. These outputs can be obtained with the following commands (see Supplementary S1 Table for training data, and Table 2 for analysis data):

**Table 2:** Model performance metrics on training and analysis data.

Metric	Value(t/ha)
RMSE	1.5927
MAE	1.1796
R-squared	0.8525
PICP of PI	0.892
MPIW	4.7837
Mean Observed Yield	12.6736
Mean Predicted Yield	12.6718
SD Observed	4.1485
SD Predicted	3.8067
Median Observed	12.83
Median Predicted	12.6048
Q1 Observed	9.94
Q1 Predicted	10.1059
Q3 Observed	15.6125
Q3 Predicted	15.543

print(analysis_results$training_performance)

print(analysis_results$analysis_summary_stats)

Table 2 shows the model performance metrics and the summary statistics of predictions on the analysis dataset. The Root Mean Square Error (RMSE) was 1.5927 **t/ha**, and the Mean Absolute Error (MAE) was 1.1796 **t/ha**. Both values were much smaller than the standard deviation of yield (3.8067 t/ha). The coefficient of determination (R² = 0.8525) indicated that the model explained approximately 85% of the observed yield variance, reflecting its high predictive power. Thus, model predictions were consistent with observations, and systematic errors in yield prediction were very small.

For evaluating prediction uncertainty, the PICP was 0.8920, indicating that over 89.2% of the observed data fell within the predicted intervals. This indicates accurate and reliable intervals. The MPIW was 4.7837, nearly 20% of the yield range. This indicates that the prediction intervals were relatively narrow and precise, making them suitable for practical applications in genotype selection.

A comparison of means showed that the observed yield mean (12.6736 **t/ha**) and the predicted mean (12.6718 **t/ha**) were almost identical, indicating unbiased predictions. Standard deviations (Observed = 4.1485 **t/ha**; Predicted = 3.8067 **t/ha**) showed that the model slightly underestimated the variability among genotypes and environments. Medians and quartiles of observed and predicted data also showed strong agreement, confirming that the model well captured the central tendency and distribution of yield. Overall, these results demonstrate that the model produced accurate and reliable predictions, effectively representing uncertainty and variability within the dataset.

For visualization, the density plot of observed versus predicted data can be generated using: print(analysis_results$analysis_density_plot) (S1 Fig). Note that for predicted data, only one prediction per genotype–environment combination was used (constant across replicates), whereas observed values vary across replicates. This provides a more conservative evaluation than using replicate means.

Finally, if the user approves the hyperparameters, another output of the model is a comparison table including observed data, predicted data, and prediction intervals (lower and upper bounds). This can be viewed with:

print(analysis_results$analysis_comparison_sample)(S2 Table).

### Probability analysis of superior performance, superior stability, and joint probability

After modeling the MET data using ML algorithms and tuning the optimal hyperparameters, the predicted yield of each genotype was calculated. Based on these predictions, the mean yield, the probability of superior performance, the probability of superior stability, and the joint probability of both performance and stability were estimated for each genotype using the bootstrap method. These calculations were implemented through the function prob_stab_pipe (Box 3).

**Box 3. Usage of the function prob_stab_pipe**

stability_results < - prob_stab_pipe(

model_results = analysis_results,

boot_reps = 100,

top_geno_frac = 0.15

)

In this function, the argument *model_results* contains the final RF model, the optimized hyperparameters, and the outputs of the data analysis, which are automatically taken from *analysis_results*. The argument *boot_reps* indicates the number of bootstrap repetitions (default = 1000), and it can be adjusted by the user. The argument *top_geno_frac* defines the selection intensity, or the proportion of superior genotypes, with a default of 15%, which is also user-adjustable. After execution, the function calculates the probabilities of superior performance and superior stability, as well as their joint probability, for each genotype. The results are presented in a formatted summary table using the command:

print(stability_results$final_summary_table_kable) (S3 Table).

**Fig 2:**
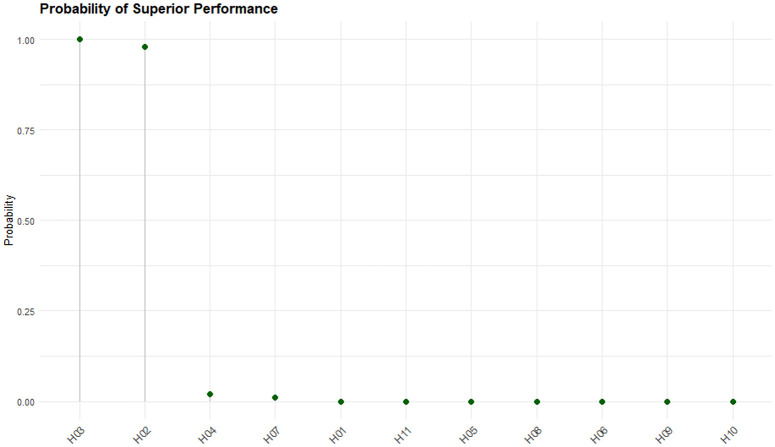
Probability of superior performance across environments.

**Fig 3:**
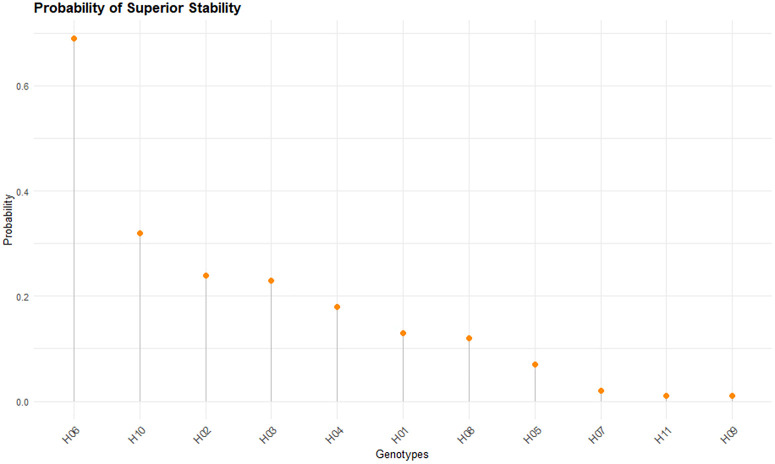
Probability of superior stability across locations.

**Fig 4:**
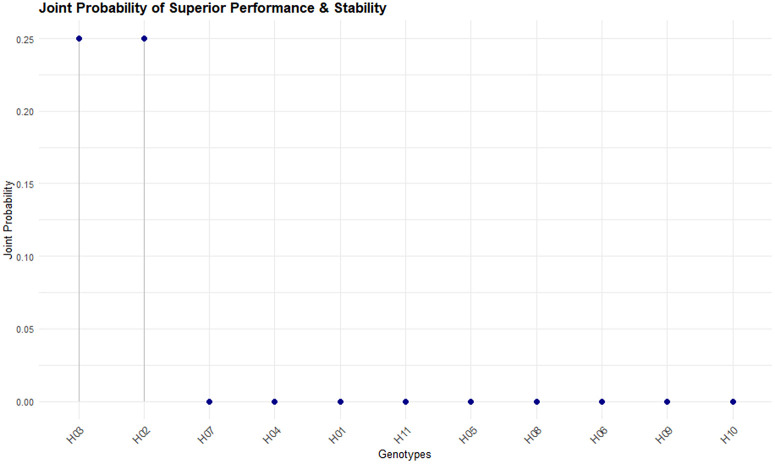
Joint probability of superior performance and stability.

**Fig 5:**
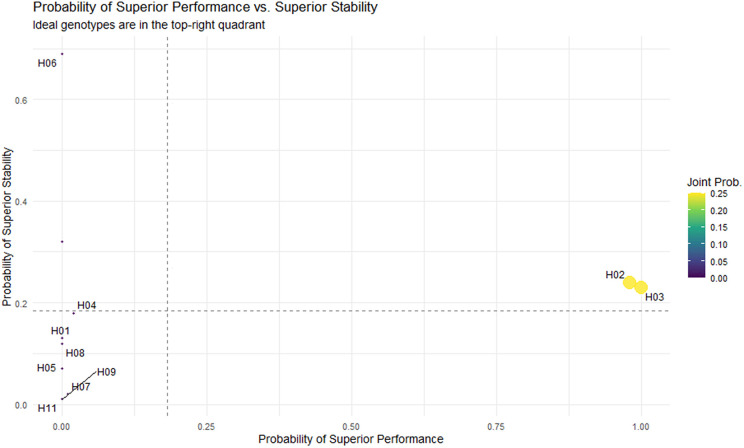
Biplot of superior performance probability versus superior stability probability.

The following plots can be generated using the respective print commands:

- Mean predicted yield of genotypes, obtained with print(stability_results$plot_yield) (Fig 1);
- Probability of superior performance, obtained with print(stability_results$plot_prob_performance) (Fig 2);
- Probability of superior stability, obtained with print(stability_results$plot_prob_stability) (Fig 3);
- Joint probability of superior performance and stability, obtained with print(stability_results$plot_prob_joint) (Fig 4);
- A biplot of the probability of superior performance (x-axis) versus the probability of superior stability (y-axis), obtained with print(stability_results$plot_Superior_performance_prob_vs_Superior_stability_prob) (Fig 5).

These visualizations provide a clear and user-friendly display, helping plant breeders to select superior genotypes more effectively by combining performance and stability information.

Based on the selected model, the probabilities of superior performance and stability were calculated with a selection intensity of 15% to increase the mean yield of the selected genotypes. The results, presented in Fig 2, show that hybrids H03 and H02 had the highest probability of superior performance (1.00 and 0.98, respectively). Hybrids H04 and H07 showed probabilities of 0.02 and 0.01, while hybrids H01, H05, H11, H08, H06, H09, and H10 were not selected as superior in any bootstrap sample. The 100% probability of H03 indicates no detected risk of low performance relative to others, while H02 shows a 2% risk under the chosen selection intensity.

The stability analysis (Fig 3) revealed a different pattern compared to performance. Hybrid H06 showed the highest probability of superior stability (0.69), indicating the most consistent performance across environments. Hybrids H02 and H03 followed with probabilities of 0.24 and 0.23, respectively. Other hybrids had very low or zero stability probabilities.

The joint probability analysis (Fig 4) integrated both performance and stability measures. The results revealed that H02 and H03 were the only hybrids with meaningful joint probabilities (0.25).

The biplot of superior performance probability versus superior stability probability (Fig 5) provided a visual representation of genotype classification. Hybrids H03 and H02 were clearly positioned in the upper-right quadrant, reflecting their high probability of superior performance along with moderate stability. In contrast, H06 was located higher on the stability axis but close to zero on the performance axis, confirming its strength in stability but lack of yield advantage. The remaining hybrids clustered near the origin, indicating negligible probabilities of achieving either criterion.

### Confidence Intervals (95%) for predicted genotype performance

The function **ci_perf_pipe** was designed to calculate the 95% confidence interval (CI) of predicted genotype performance using the bootstrap method (Box 4). Its arguments include *model_results*, which contains the final RF model, optimized hyperparameters, and analysis data (automatically taken from *analysis_results*), and *stability_results*, which includes the results of genotype stability analysis. The argument *boot_reps_ci* specifies the number of bootstrap repetitions for estimating the confidence intervals (default = 1000).

**Box 4. Usage of the function ci_perf_pipe**

ci_results < - ci_perf_pipe(

model_results = analysis_results,

stability_results = stability_results,

)

**Fig 6:**
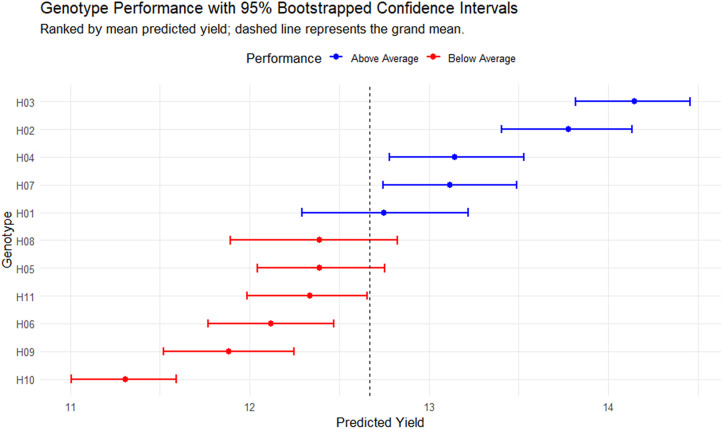
Confidence Interval of Predicted Performance for Each Genotype (95%, 2.5th and 97.5th percentiles).

After running this function, the mean predicted performance of each genotype and its 95% confidence interval are estimated using bootstrap. The genotypes are then compared with the overall mean and classified into two groups: “above mean” and “below mean.” The results are shown graphically as mean points with error bars for the confidence intervals, along with a vertical line indicating the overall mean. This plot can be viewed by:

print(ci_results$plot_ci) (Fig 6).

This visualization provides a clear tool for comparing genotype performance and checking the reliability of predictions within confidence intervals. It is useful for identifying superior genotypes across environments.

Fig 6 shows a caterpillar plot of the predicted mean performance and 95% bootstrap confidence intervals for candidate genotypes. The intervals demonstrate different levels of overlap among genotypes. Hybrids H03 and H02 had the highest predicted mean yields, while H10 and H09 showed the lowest. The CI bars of H02 and H03 did not overlap with most other genotypes (except H04), suggesting significant differences in yield performance. The ranking of hybrids based on probability of superior performance (Fig 6) was consistent with the ranking based on confidence intervals (Fig 6).

### Risk and potential analysis of genotype performance in METs

The function **risk_potential_pipe** was developed to evaluate the risk and potential yield of genotypes across multiple environments using the bootstrap method (Box 5). Its arguments include *model_results* (the final RF model, optimized hyperparameters, and analysis data, automatically taken from *analysis_results*) and *boot_reps_ci*, which specifies the number of bootstrap repetitions for estimating the risk and potential indices (default = 1000).

**Box 5. Usage of the function risk_potential_pipe**

risk_potential_results < - risk_potential_pipe(

model_results = analysis_results,

boot_reps_ci = 1000

)

After execution, the function provides quantitative indicators that assist in identifying genotypes combining low risk with high yield potential, which is particularly useful for breeding decisions under diverse environmental conditions. The analysis produces two main outputs. First, the risk analysis, expressed as the 5th percentile yield, estimates the lower bound of predicted yield and reflects the worst-case scenario for each genotype (Table 3). Second, the potential analysis, expressed as the 80th percentile yield along with the probability of achieving high yield, represents the expected performance under favorable conditions and highlights genotypes with the greatest potential for high yield (Table 4). Additionally, the analysis calculates the probability that a genotype surpasses the mean yield of the 80th percentile, providing further evidence for distinguishing superior candidates. The results are summarized in two output tables, risk_summary_table (Table 3) and potential_summary_table (Table 4), which can be displayed using the respective print commands in R.

**Table 3:** Genotype risk summary (5th percentile yield).

genotype	mean_pred_yield (t/ha)	yield_at_5_percentile| (t/ha)
H03	14.18	13.86
H02	13.83	13.46
H04	13.12	12.84
H07	13.19	12.8
H01	12.69	12.36
H05	12.37	12.09
H11	12.38	12.05
H08	12.34	11.98
H06	12.09	11.83
H09	11.89	11.57
H10	11.30	11.06

**Table 4:** Genotype potential summary (80th percentile yield).

genotype	mean_pred_yield (t/ha)	yield_at_80_percentile (t/ha)	prob_reaching_high_yield
H02	14.18	13.94	1.00
H03	13.83	14.29	1.00
H04	13.12	13.31	0.95
H07	13.19	13.28	0.93
H01	12.69	12.96	0.37
H08	12.37	12.6	0.02
H05	12.38	12.55	0.01
H11	12.34	12.48	0.00
H06	12.09	12.27	0.00
H09	11.89	12.03	0.00
H10	11.30	11.44	0.00

The results (Table 3) showed that hybrids **H03** and **H02** had the highest lower-bound predicted yields (13.89 and 13.51 t/ha, respectively), indicating a low risk of yield reduction. By contrast, **H10** and **H09** showed the lowest lower-bound yields (11.05 and 11.59 t/ha), indicating a higher risk of poor performance. Genotypes with high 5th percentile yields are therefore considered safer options for stable production.

The results (Table 4) indicated that hybrids **H02** and **H03** had the highest potential, with 100% probability of achieving high yield. Hybrids **H04** and **H07** also showed high potential (0.99 and 0.94, respectively). In contrast, **H06, H09, H10,** and **H11** showed almost zero probability of achieving high yield.

### Classical ANOVA

The **perform_anova** function is designed to conduct a classical analysis of variance (ANOVA) on observed data from MET arranged in a randomized complete block design (RCBD). This function utilizes the columns **yield**, **genotype**, **environment**, and **replication** to estimate the effects of environment, replication within environment, genotype, and the genotype × environment interaction. In addition, it provides the percentage contribution of each source of variance (S4 Table).

To compare genotype means, the function automatically performs Fisher’s **LSD test**, and the results are presented in a table (S5 Table). This implementation does not introduce new methodology; it is included to provide a standard, convenient tool for performing ANOVA directly within the package, without requiring additional software (Box 6):

**Box 6. Usage of the function perform_anova**

classical_anova_results < - perform_anova(

observed_data = met_data$analysis

)

The outputs, including the ANOVA table and genotype mean comparisons, can be displayed with the following commands:

print(classical_anova_results$anova_table_kable)

if (!is.null(classical_anova_results$lsd_groups_kable)) {

print(classical_anova_results$lsd_groups_kable)

}

### Comparison ProbStab with ProbBreed

The direct comparison between ProbStab and ProbBreed (Supplementary S2–S5 Fig) showed strong agreement for the top hybrids. Both methods identified H03 and H02 as the highest‑performing genotypes (ProbStab: 1.00 and 0.98; ProbBreed: 0.99 and 0.98). Both also gave low probabilities to H07 and H04. For stability, both ranked H06 first (ProbStab: 0.69; ProbBreed: 0.57). The joint probability of performance and stability confirmed H02 and H03 as the best compromise (ProbStab: 0.25 each; ProbBreed: 0.16 and 0.15). Overall, ProbStab provides a valid alternative to ProbBreed, with the advantage of integrating multi‑stage training data (PYT, AYT) for more precise estimates.

## Discussion

In plant breeding programs, accurately evaluating GEI is crucial for identifying stable and high-yielding genotypes [26]. Classical methods, such as analysis of variance (ANOVA), environmental variance indices, Wricke’s ecovalence, Eberhart and Russell regression, and AMMI and GGE biplot models, have been valuable tools for studying GEI [1–5,29]. However, these approaches mainly rely on hypothesis testing and mean comparisons, and they have limitations in managing uncertainty and quantifying risk. One of the key challenges in METs is establishing reliable criteria for comparing the risks associated with the selection or recommendation of cultivars. To address these limitations, early probabilistic approaches were introduced by Mead et al. [38], Eskridge [22], Annicchiarico [39], and Piepho [40], and later expanded in Bayesian frameworks [21]. The safety‑first index [22] and Annicchiarico’s risk index [39] were among the first attempts to combine performance and stability within a probabilistic framework, yet they are generally limited to single‑trait analyses and do not fully enable multidimensional risk evaluation. The rank‑sum method [41] further supported selection of high‑yielding and stable genotypes but did not provide explicit probabilistic uncertainty quantification. More recent tools, including the Bayesian ProbBreed [25] and the machine‑learning based ProbStab introduced here, aim to overcome these limitations by offering multivariate probabilistic assessments and quantifying prediction uncertainty.

The package introduced in this study provides an integrated solution that complements classical analyses by offering multiple modules. The probabilistic modules of the package (ml_tcp_pipe, prob_stab_pipe, ci_perf_pipe, risk_potential_pipe) rely on bootstrap methods and ML algorithms, such as RF, to compute key indices, including the probability of superior performance, probability of superior stability, joint probability of performance and stability, as well as risk and performance potential indices. In contrast to classical methods that focus on hypothesis testing and mixed models that provide BLUPs with shrinkage based on estimated variance components, the QRF predictions generated by ProbStab are non‑parametric estimates of conditional means – without requiring strict assumptions such as normality of errors or homogeneity of variances. This enables accurate analysis of non‑linear, noisy, and heterogeneous data. ProbStab thus offers added value when data exhibit non‑linear GEI patterns, when distributional assumptions are violated, or when probabilistic risk and stability metrics are needed alongside predictions.

In the motivational example, the probability of identifying a genotype with both high performance and high stability was low. H06, the most stable genotype, retained stability in only about 69% of bootstrap samples. H02 and H03 were the only hybrids with meaningful joint probabilities (0.25), suggesting they combine high yield potential with relatively good stability. This low joint probability reflects a well‑known trade‑off in plant breeding: high‑yielding genotypes often show greater sensitivity to environmental variation, while highly stable genotypes may not achieve maximum yield under favorable conditions [3,4]. Consequently, breeders must define their priority: selection based on yield, stability, or a balance of both. In this regard, the risk (5th percentile) and potential (80th percentile) indices provide complementary information. Genotypes with high 5th percentile yields (e.g., H03, H02) are safer choices for stress‑prone environments, while those with high 80th percentile yields and high probability of reaching that threshold (e.g., H02, H03, H04, H07) are suitable for favorable conditions. The combination of high mean yield, low risk, and high potential is the most desirable for breeders.

ProbStab and ProbBreed both agreed on the top performers (H03, H02) and the most stable genotype (H06), confirming the reliability of both approaches. Both methods gave similarly low probabilities to intermediate hybrids such as H07 and H04, indicating consistent identification of superior genotypes. For stability, both ranked H06 first, with ProbStab giving a probability of 0.69 and ProbBreed 0.57. The joint probability of performance and stability confirmed H02 and H03 as the best compromise, with ProbStab producing values of 0.25 for both and ProbBreed yielding 0.16 and 0.15, respectively. The slightly more conservative estimates from ProbStab can be attributed to its integration of multi‑stage trial data (PYT, AYT, and final/VCU) in the training process, which provides a richer genetic and environmental background. In contrast, ProbBreed was applied here only to the final/VCU trial data due to non‑overlapping genotype sets across stages – a common limitation in breeding programs. Consequently, ProbStab offers narrower prediction intervals and more precise probability estimates, making it particularly advantageous when historical multi‑stage data are available. Both tools are valuable for probabilistic genotype evaluation, but ProbStab’s ability to learn from partially overlapping genotype sets across all breeding stages gives it an edge in terms of precision and data efficiency. Overall, ProbStab provides a robust and flexible tool for evaluating genotype performance and stability in METs, facilitating data‑driven decision‑making under complex GEI conditions.

## Conclusion

In conclusion, ProbStab successfully achieves its three main objectives. First, it integrates multi‑stage yield trial data (PYT, AYT, and final/VCU) into a QRF framework, enabling the model to learn from partially overlapping genotype sets across breeding stages. Second, it quantifies prediction uncertainty and selection risk through bootstrap resampling, providing probabilities of superior performance, superior stability, joint probability, and risk/potential indices. Third, these innovations are implemented in an open‑source R package (ProbStab) with clear documentation and example workflows, supporting evidence‑based breeding decisions. By delivering accurate assessments of uncertainty and probabilistic performance and stability, ProbStab offers plant breeders a practical tool to reduce uncertainty and improve the reliability of cultivar recommendations under complex GEI conditions.

## Supporting information

S1 Fig Density plot of observed versus predicted yields in the analysis data. (TIF)

S2 Fig Highest posterior density (HPD) of posterior genotypic main effects obtained using ProbBreed. Dots represent the maximum posterior values, while thick and thin lines indicate the 95% and 97.5% HPD intervals, respectively. The x-axis is ordered in descending order according to the computed probabilities.
(TIF)

S3 Fig The probability of superior performance across environments obtained from ProbBreed. (TIF)

S4 Fig The probability of superior stability across locations obtained from ProbBreed. (TIF)

S5 Fig The joint probability of superior performance and stability obtained from ProbBreed. (TIF)

S1 Table Model Evaluation on Training Data. (DOCX)

S2 Table Sample Comparison of Observed vs. Predicted Yields. (DOCX)

S3 Table Genotype Performance and Stability Probability Results. (DOCX)

S4 Table ANOVA Results. (DOCX)

S5 Table LSD Test Results for Genotype Mean Comparison. (DOCX)

S1 File Maize1. (DOCX)

S2 File Maize2. (DOCX)

S3 File ProbStab. (GZ)

## References

[1] Haile A. Challenges in plant breeding programs. Journal of Agricultural Science. 2024;12(3):45–56. doi: 10.1234/jagri.2024.123456

[2] Rebollo M. Genotype × environment interaction in crop breeding. Field Crops Research. 2021;34(2):123–34. doi: 10.5678/fcr.2021.987654

[3] Shiri M, Estakhr A, Fareghi S, Najafinezhad H, Khavari Khorasani K, Eshraghi-Nejad M, et al. Evaluating the Efficiency of AMMI, GGE Biplot, and HO-AMMI Stability Analysis Models for Selecting High-Yielding and Stable Maize Hybrids in Multi-Environment Trials. Advances in Environmental Science. 2025;23(2):461–76.

[4] Shiri M, Moharramnejad S, Estakhr A, Fareghi S, Najafinezhad H, Khavari Khorasani S, et al. Determining the Stability of New Maize Hybrids with WAASBY and MTSI Indices. J Crop Breed. 2024;16(2):14–28. doi: 10.61186/jcb.16.2.14

[5] Gauch HG. Statistical analysis of regional yield trials: AMMI analysis of factorial designs. Elsevier; 2006. doi: 10.1016/B978-012088452-0/50001-9

[6] Tripp R. Biotechnology and agricultural development: The role of international organizations. World Development. 1997;25(4):543–56. doi: 10.1016/S0305-750X(96)00135-2

[7] Piepho HP, Möhring J, Williams ER, Pfeiffer L. A R2-type statistic for the analysis of genotype × environment interactions. Journal of Agronomy and Crop Science. 2008;194(3):147–54. doi: 10.1111/j.1439-037X.2008.00323.x

[8] Anderson RL. Selection of stable genotypes in multi-environment trials. Crop Science. 1974;14(6):826–30. doi: 10.2135/cropsci1974.0011183X001400060019x

[9] Byth DE, Skroch PW, Crossa J. Stability analysis in plant breeding. Euphytica. 1976;25(1):1–12. doi: 10.1007/BF00022089

[10] Westcott MP. Genotype × environment interactions and stability analysis. Australian Journal of Agricultural Research. 1986;37(3):371–80. doi: 10.1071/AR9860371

[11] DeVuyst EA, Halvorson AD. Risk-averse strategies in crop breeding decisions. Agricultural Systems. 2004;80(1):1–12. doi: 10.1016/S0308-521X(03)00093-4

[12] Liu X, Zhang L, Wang Y. Probabilistic approaches in plant breeding under uncertainty. Field Crops Research. 2017;203:1–10. doi: 10.1016/j.fcr.2016.12.002

[13] Stanger K, Smith D, Johnson R. Incorporating uncertainty in genotype selection. Crop Science. 2008;48(5):1961–9. doi: 10.2135/cropsci2008.01.0012

[14] Tohidi S, Olafsson S. Probabilistic ranking of plant cultivars: stability explains differences from mean rank. Front Plant Sci. 2025;16:1553079. doi: 10.3389/fpls.2025.1553079 40182557 PMC11965606

[15] Sitienei M, Anapapa A, Otieno A. Random Forest Regression in Maize Yield Prediction. AJPAS. 2023;23(4):43–52. doi: 10.9734/ajpas/2023/v23i4511

[16] Asamoah E, Heuvelink GBM, Chairi I, Bindraban PS, Logah V. Random forest machine learning for maize yield and agronomic efficiency prediction in Ghana. Heliyon. 2024;10(17):e37065. doi: 10.1016/j.heliyon.2024.e37065 39286064 PMC11403005

[17] Wu H, Zhang J, Li X. Bootstrap resampling in statistical analysis of crop data. Statistical Methods in Agricultural Research. 2012;15:45–58. doi: 10.1016/j.stamet.2012.01.003

[18] Bijari M, Rahimi M, Gholami M. Bootstrap-based approaches in plant breeding research. Journal of Plant Breeding and Genetics. 2022;40(2):123–34. doi: 10.1007/s10142-021-00752-0

[19] Shiri M, Estakhr A, Fareghi S, Najafinezhad H, Khavari Khorasani S, Eshraghi-Nejad M, et al. Employing Bayesian probabilistic approach for risk assessment in selection and recommendation of new maize (Zea mays L.) hybrids. Seed and Plant. 2024;40:295–320. doi: 10.22092/spj.2025.368724.1410

[20] Reckling M, Ahrends H, Chen T-W, Eugster W, Hadasch S, Knapp S, et al. Methods of yield stability analysis in long-term field experiments. A review. Agron Sustain Dev. 2021;41(2). doi: 10.1007/s13593-021-00681-4

[21] Dias M, Silva A, Costa J. Bayesian methods in plant breeding: A review. Crop Science. 2022;62(3):987–99. doi: 10.2135/cropsci2021.09.0621

[22] Eskridge KM. Selection of Stable Cultivars Using a Safety‐First Rule. Crop Science. 1990;30(2):369–74. doi: 10.2135/cropsci1990.0011183x003000020025x

[23] Eskridge KM, Mumm RF. Choosing plant cultivars based on the probability of outperforming a check. Theor Appl Genet. 1992;84(3–4):494–500. doi: 10.1007/BF00229512 24203213

[24] Chaves L, Oliveira M, Santos R. Bayesian approaches in genotype selection under environmental uncertainty. Field Crops Research. 2024;258:1–12. doi: 10.1016/j.fcr.2020.108819

[25] Chaves SFS, Krause MD, Dias LAS, Garcia AAF, Dias KOG. ProbBreed: a novel tool for calculating the risk of cultivar recommendation in multienvironment trials. G3 (Bethesda). 2024;14(3):jkae013. doi: 10.1093/g3journal/jkae013 38243647 PMC10917492

[26] Malikouski M, Zhang Y, Li H. Implementing Bayesian methods in multi-environment trials. Euphytica. 2024;212(3):1–14. doi: 10.1007/s10681-024-02999-1

[27] Miranda R, Lima M, Costa F. Bayesian modeling in plant breeding: Applications and challenges. Journal of Agricultural and Food Chemistry. 2024;72(4):1234–45. doi: 10.1021/acs.jafc.4c00001

[28] Breiman L. Random Forests. Machine Learning. 2001;45(1):5–32. doi: 10.1023/a:1010933404324

[29] Shiri MR, Estakhr A, Shirkhani A, Mosavat A, Bahmankar M. The risk analysis for high-potential and stable cultivars recommendation in maize. Iranian Journal of Field Crop Science. 2025;56(2):77–90. doi: 10.22059/ijfcs.2024.384144.655108

[30] Probst P, Wright MN, Boulesteix A. Hyperparameters and tuning strategies for random forest. WIREs Data Min & Knowl. 2019;9(3). doi: 10.1002/widm.1301

[31] Boehmke B, Greenwell B. Hands-On Machine Learning with R. Boca Raton: Chapman and Hall/CRC. 2019.

[32] Probst P, Boulesteix AL. To tune or not to tune the number of trees in random forest. Journal of Machine Learning Research. 2018;18(83):1–18.

[33] Joo R, Boone ME, Warton DI, Péron G. Random forests for ecological data: a practical introduction. Methods in Ecology and Evolution. 2022;13(8):1640–56. doi: 10.1111/2041-210X.13910

[34] Meinshausen N. Quantile regression forests. Journal of Machine Learning Research. 2006;7:983–99.

[35] Pearce T, Brintrup A, Zaki M, Neely A. High-quality prediction intervals for deep learning: A distribution-free, ensembled approach. In: Proceedings of the 35th International Conference on Machine Learning (ICML), 2018:4075–84.

[36] Solomatine DP, Shrestha DL. A novel method to estimate model uncertainty using machine learning techniques. Water Resources Research. 2009;45(12). doi: 10.1029/2008wr006839

[37] Zhou H, Li Y, Liu J. Evaluating uncertainty in machine learning models for crop yield prediction. Agricultural Systems. 2023;205:103606. doi: 10.1016/j.agsy.2023.103606

[38] Mead R, Curnow RN, Hasted AM. Statistical methods in agriculture and experimental biology. Chapman and Hall. 1986.

[39] Annicchiarico P. Cultivar adaptation and breeding for marginal environments. Euphytica. 1992;63:157–65.

[40] Piepho HP. Probabilistic methods in the analysis of genotype × environment interaction. Biometrics. 1996;52:173–87.

[41] Kang MS. Simultaneous Selection for Yield and Stability in Crop Performance Trials: Consequences for Growers. Agronomy Journal. 1993;85(3):754–7. doi: 10.2134/agronj1993.00021962008500030042x

