# Supplementary material for: ProbStab: A probabilistic ML-assisted pipeline for genotype performance, stability, and risk evaluation in multi-environment trials
Source: PLoS One. 2026 Jul 10;21(7):e0352098. doi: 10.1371/journal.pone.0352098 (PMC13354077; doi:10.1371/journal.pone.0352098)
Supplement: S1 Table — (DOCX) [file pone.0352098.s006.docx]

**Table S1: Table: Model Evaluation on Training Data**

| Metric | Value |
| --- | --- |
| RMSE | 1.4320 |
| MAE | 1.0512 |
| R-squared | 0.8873 |
| \|PICP of PI | 0.9122 |
