# Supplementary material for: ProbStab: A probabilistic ML-assisted pipeline for genotype performance, stability, and risk evaluation in multi-environment trials
Source: PLoS One. 2026 Jul 10;21(7):e0352098. doi: 10.1371/journal.pone.0352098 (PMC13354077; doi:10.1371/journal.pone.0352098)
Supplement: S2 Table — (DOCX) [file pone.0352098.s007.docx]

Table S2: Sample Comparison of Observed vs. Predicted Yields

| environment | genotype | rep | yield | predicted_yield | lower_pi | upper_pi |
| --- | --- | --- | --- | --- | --- | --- |
| E1.3 | H01 | 1 | 14.45 | 15.47 | 13.06 | 17.84 |
| E1.3 | H02 | 1 | 14.16 | 14.64 | 12.22 | 17.01 |
| E1.3 | H03 | 1 | 15.57 | 14.07 | 11.65 | 16.44 |
| E1.3 | H04 | 1 | 14.56 | 15.12 | 12.71 | 17.49 |
| E1.3 | H05 | 1 | 15.23 | 14.85 | 12.43 | 17.21 |
| E1.3 | H06 | 1 | 13.49 | 14.51 | 12.09 | 16.88 |
| E1.3 | H07 | 1 | 12.52 | 14.37 | 11.95 | 16.73 |
| E1.3 | H08 | 1 | 14.52 | 16.07 | 13.66 | 18.44 |
| E1.3 | H09 | 1 | 11.05 | 13.66 | 11.24 | 16.02 |
| E1.3 | H10 | 1 | 11.49 | 14.41 | 11.99 | 16.78 |
| E1.3 | H11 | 1 | 12.97 | 14.43 | 12.01 | 16.8 |
| E1.3 | H06 | 2 | 13.2 | 14.51 | 12.09 | 16.88 |
| E1.3 | H02 | 2 | 13.96 | 14.64 | 12.22 | 17.01 |
| E1.3 | H09 | 2 | 13.74 | 13.66 | 11.24 | 16.02 |
| E1.3 | H10 | 2 | 15.06 | 14.41 | 11.99 | 16.78 |
