# Supplementary material for: ProbStab: A probabilistic ML-assisted pipeline for genotype performance, stability, and risk evaluation in multi-environment trials
Source: PLoS One. 2026 Jul 10;21(7):e0352098. doi: 10.1371/journal.pone.0352098 (PMC13354077; doi:10.1371/journal.pone.0352098)
Supplement: S3 Table — (DOCX) [file pone.0352098.s008.docx]

Table S3: Genotype Performance and Stability Probability Results

| genotype | mean_pred_  yield | Superior_performance  _prob | Superior_stability  _prob | Joint_superior_perf_  stability_prob |
| --- | --- | --- | --- | --- |
| H03 | 14.18 | 1 | 0.23 | 0.25 |
| H02 | 13.83 | 0.98 | 0.24 | 0.25 |
| H07 | 13.19 | 0.01 | 0.02 | 0 |
| H04 | 13.12 | 0.02 | 0.18 | 0 |
| H01 | 12.69 | 0 | 0.13 | 0 |
| H11 | 12.38 | 0 | 0.01 | 0 |
| H05 | 12.37 | 0 | 0.07 | 0 |
| H08 | 12.34 | 0 | 0.12 | 0 |
| H06 | 12.09 | 0 | 0.69 | 0 |
| H09 | 11.89 | 0 | 0.01 | 0 |
| H10 | 11.3 | 0 | 0.32 | 0 |
