# Supplementary material for: ProbStab: A probabilistic ML-assisted pipeline for genotype performance, stability, and risk evaluation in multi-environment trials
Source: PLoS One. 2026 Jul 10;21(7):e0352098. doi: 10.1371/journal.pone.0352098 (PMC13354077; doi:10.1371/journal.pone.0352098)
Supplement: S4 Table — (DOCX) [file pone.0352098.s009.docx]

Table S4: ANOVA Results

| Source | Df | Sum Sq | Mean Sq | F value | Pr(>F) | % Variation |
| --- | --- | --- | --- | --- | --- | --- |
| environment | 19 | 10933.79 | 575.4628 | 201.9565 | 0.0000 | 84.7286 |
| Replication within E | 60 | 513.2147 | 8.5536 | 3.0018 | 0.0000 | NA |
| genotype | 10 | 590.1033 | 59.0103 | 20.7095 | 0.0000 | 4.5729 |
| environment:genotype | 190 | 1380.591 | 7.2663 | 2.5501 | 0.0000 | 10.6985 |
| Residuals | 600 | 1709.663 | 2.8494 | NA | NA | NA |
