# Supplementary material for: ProbStab: A probabilistic ML-assisted pipeline for genotype performance, stability, and risk evaluation in multi-environment trials
Source: PLoS One. 2026 Jul 10;21(7):e0352098. doi: 10.1371/journal.pone.0352098 (PMC13354077; doi:10.1371/journal.pone.0352098)
Supplement: S5 Table — (DOCX) [file pone.0352098.s010.docx]

Table S5: LSD Test Results for Genotype Mean Comparison

| genotype | yield | groups |
| --- | --- | --- |
| H03 | 14.20963 | a |
| H02 | 13.825 | a |
| H07 | 13.23588 | b |
| H04 | 13.10525 | bc |
| H01 | 12.67437 | cd |
| H11 | 12.41938 | de |
| H05 | 12.36663 | de |
| H08 | 12.31462 | de |
| H06 | 12.07662 | e |
| H09 | 11.90713 | e |
| H10 | 11.27463 | f |
