# Supplementary material for: ProbStab: A probabilistic ML-assisted pipeline for genotype performance, stability, and risk evaluation in multi-environment trials
Source: PLoS One. 2026 Jul 10;21(7):e0352098. doi: 10.1371/journal.pone.0352098 (PMC13354077; doi:10.1371/journal.pone.0352098)
Supplement: S1 File — (DOCX) [file pone.0352098.s011.docx]

Environment Rep Genotype Yield

E1.1 1 H96 7.05

E1.1 1 H117 8.64

E1.1 1 H34 13.22

E1.1 1 H23 10.16

E1.1 1 H14 12.03

E1.1 1 H123 14.01

E1.1 1 H132 11.89

E1.1 1 H77 13.91

E1.1 1 H106 13.83

E1.1 1 H139 11.90

E1.1 1 H11 14.80

E1.1 1 H87 12.09

E1.1 1 H31 10.51

E1.1 1 H27 11.34

E1.1 1 H64 14.61

E1.1 1 H61 15.17

E1.1 1 H88 14.37

E1.1 1 H93 11.91

E1.1 1 H45 15.62

E1.1 1 H55 14.94

E1.1 1 H146 10.09

E1.1 1 H98 6.68

E1.1 1 H84 11.26

E1.1 1 H125 11.93

E1.1 1 H110 6.25

E1.1 1 H50 13.09

E1.1 1 H144 8.16

E1.1 1 H103 10.10

E1.1 1 H48 12.18

E1.1 1 H71 12.13

E1.1 1 H80 10.41

E1.1 1 H42 7.57

E1.1 1 H141 13.75

E1.1 1 H28 8.57

E1.1 1 H102 10.96

E1.1 1 H74 11.90

E1.1 1 H114 12.85

E1.1 1 H58 12.59

E1.1 1 H128 10.99

E1.1 1 H68 11.20

E1.1 1 H118 13.12

E1.1 1 H01 9.56

E1.1 1 H04 11.56

E1.1 1 H17 15.78

E1.1 1 H10 9.12

E1.1 1 H41 12.13

E1.1 1 H95 10.31

E1.1 1 H76 11.20

E1.1 1 H67 13.92

E1.1 1 H140 10.98

E1.1 1 H57 13.87

E1.1 1 H36 15.06

E1.1 1 H101 9.70

E1.1 1 H79 12.10

E1.1 1 H06 16.68

E1.1 1 H26 9.06

E1.1 1 H39 14.57

E1.1 1 H97 14.28

E1.1 1 H70 13.75

E1.1 1 H13 15.64

E1.1 1 H130 10.16

E1.1 1 H30 14.85

E1.1 1 H22 10.37

E1.1 1 H47 2.84

E1.1 1 H120 11.22

E1.1 1 H16 16.71

E1.1 1 H21 10.44

E1.1 1 H63 12.37

E1.1 1 H33 10.12

E1.1 1 H122 11.83

E1.1 1 H19 14.29

E1.1 1 H143 10.34

E1.1 1 H108 7.79

E1.1 1 H83 13.65

E1.1 1 H124 8.56

E1.1 1 H131 13.16

E1.1 1 H113 13.36

E1.1 1 H02 15.51

E1.1 1 H92 10.86

E1.1 1 H86 12.84

E1.1 1 H109 10.45

E1.1 1 H105 12.14

E1.1 1 H90 13.29

E1.1 1 H53 13.08

E1.1 1 H66 10.49

E1.1 1 H116 12.91

E1.1 1 H127 8.59

E1.1 1 H15 9.99

E1.1 1 H40 10.66

E1.1 1 H51 12.21

E1.1 1 H121 11.94

E1.1 1 H107 16.15

E1.1 1 H69 12.10

E1.1 1 H112 12.59

E1.1 1 H94 15.09

E1.1 1 H20 12.11

E1.1 1 H56 18.92

E1.1 1 H43 14.56

E1.1 1 H129 8.07

E1.1 1 H126 12.79

E1.1 1 H111 14.74

E1.1 1 H62 14.69

E1.1 1 H24 11.50

E1.1 1 H91 14.91

E1.1 1 H89 15.10

E1.1 1 H03 17.96

E1.1 1 H82 11.46

E1.1 1 H12 14.32

E1.1 1 H99 15.58

E1.1 1 H115 10.86

E1.1 1 H119 7.51

E1.1 1 H145 14.13

E1.1 1 H142 19.03

E1.1 1 H135 11.16

E1.1 1 H104 14.47

E1.1 1 H35 11.90

E1.1 1 H59 11.33

E1.1 1 H100 12.85

E1.1 1 H18 19.14

E1.1 1 H32 12.30

E1.1 1 H46 12.45

E1.1 1 H25 8.50

E1.1 1 H05 14.24

E1.1 1 H81 11.40

E1.1 1 H65 12.36

E1.1 1 H78 7.91

E1.1 1 H85 8.32

E1.1 1 H75 10.23

E1.1 1 H29 10.82

E1.1 2 H119 9.99

E1.1 2 H39 11.01

E1.1 2 H92 11.42

E1.1 2 H56 13.72

E1.1 2 H48 16.29

E1.1 2 H66 15.51

E1.1 2 H139 12.21

E1.1 2 H78 14.20

E1.1 2 H105 14.05

E1.1 2 H32 14.30

E1.1 2 H109 11.08

E1.1 2 H40 13.60

E1.1 2 H107 17.27

E1.1 2 H128 9.58

E1.1 2 H27 12.57

E1.1 2 H59 13.81

E1.1 2 H67 11.28

E1.1 2 H83 13.98

E1.1 2 H01 17.59

E1.1 2 H116 14.59

E1.1 2 H75 16.24

E1.1 2 H97 13.39

E1.1 2 H71 20.75

E1.1 2 H144 9.83

E1.1 2 H29 16.52

E1.1 2 H101 14.59

E1.1 2 H96 12.72

E1.1 2 H51 14.82

E1.1 2 H132 12.64

E1.1 2 H35 13.78

E1.1 2 H43 13.31

E1.1 2 H10 11.20

E1.1 2 H88 19.11

E1.1 2 H63 13.77

E1.1 2 H141 16.47

E1.1 2 H124 9.51

E1.1 2 H81 17.22

E1.1 2 H123 14.88

E1.1 2 H112 13.77

E1.1 2 H23 7.82

E1.1 2 H86 12.74

E1.1 2 H53 13.10

E1.1 2 H146 13.79

E1.1 2 H45 13.14

E1.1 2 H104 11.33

E1.1 2 H85 11.10

E1.1 2 H140 10.69

E1.1 2 H26 11.63

E1.1 2 H90 13.45

E1.1 2 H115 13.80

E1.1 2 H126 12.11

E1.1 2 H55 13.79

E1.1 2 H122 11.52

E1.1 2 H74 13.72

E1.1 2 H50 11.09

E1.1 2 H65 11.22

E1.1 2 H69 8.80

E1.1 2 H62 11.53

E1.1 2 H70 12.84

E1.1 2 H143 13.27

E1.1 2 H100 10.71

E1.1 2 H28 9.11

E1.1 2 H131 11.83

E1.1 2 H106 10.35

E1.1 2 H114 10.98

E1.1 2 H82 12.74

E1.1 2 H02 15.75

E1.1 2 H34 11.85

E1.1 2 H80 13.95

E1.1 2 H111 11.60

E1.1 2 H130 13.78

E1.1 2 H31 13.54

E1.1 2 H18 13.52

E1.1 2 H99 9.85

E1.1 2 H42 11.86

E1.1 2 H21 7.44

E1.1 2 H127 10.69

E1.1 2 H95 8.96

E1.1 2 H22 6.74

E1.1 2 H13 13.14

E1.1 2 H47 10.76

E1.1 2 H77 9.23

E1.1 2 H15 9.67

E1.1 2 H118 7.72

E1.1 2 H91 9.15

E1.1 2 H58 9.45

E1.1 2 H20 12.24

E1.1 2 H121 13.79

E1.1 2 H05 15.63

E1.1 2 H102 12.95

E1.1 2 H17 19.12

E1.1 2 H12 18.29

E1.1 2 H113 13.75

E1.1 2 H33 11.91

E1.1 2 H120 13.52

E1.1 2 H19 16.60

E1.1 2 H68 11.62

E1.1 2 H87 11.78

E1.1 2 H93 12.74

E1.1 2 H46 12.03

E1.1 2 H129 8.80

E1.1 2 H04 17.26

E1.1 2 H16 16.08

E1.1 2 H06 15.02

E1.1 2 H64 12.12

E1.1 2 H89 9.69

E1.1 2 H57 11.19

E1.1 2 H84 9.35

E1.1 2 H103 10.57

E1.1 2 H145 9.73

E1.1 2 H76 13.76

E1.1 2 H61 13.82

E1.1 2 H108 9.26

E1.1 2 H125 13.25

E1.1 2 H24 9.19

E1.1 2 H41 11.97

E1.1 2 H79 8.18

E1.1 2 H14 12.02

E1.1 2 H03 13.02

E1.1 2 H135 9.74

E1.1 2 H94 7.89

E1.1 2 H142 9.86

E1.1 2 H25 4.58

E1.1 2 H11 8.66

E1.1 2 H110 6.23

E1.1 2 H98 9.24

E1.1 2 H36 9.16

E1.1 2 H117 7.28

E1.1 2 H30 5.93

E2.1 1 H96 12.26

E2.1 1 H117 11.87

E2.1 1 H34 11.92

E2.1 1 H23 8.53

E2.1 1 H14 12.91

E2.1 1 H123 7.95

E2.1 1 H132 8.33

E2.1 1 H77 10.27

E2.1 1 H106 8.82

E2.1 1 H139 14.94

E2.1 1 H11 9.68

E2.1 1 H87 12.59

E2.1 1 H31 11.93

E2.1 1 H27 6.29

E2.1 1 H64 7.22

E2.1 1 H61 7.03

E2.1 1 H88 9.23

E2.1 1 H93 6.86

E2.1 1 H45 6.13

E2.1 1 H55 6.99

E2.1 1 H146 6.06

E2.1 1 H98 7.43

E2.1 1 H84 7.77

E2.1 1 H125 6.86

E2.1 1 H110 5.62

E2.1 1 H50 6.28

E2.1 1 H144 4.82

E2.1 1 H103 8.35

E2.1 1 H48 8.20

E2.1 1 H71 7.77

E2.1 1 H80 7.75

E2.1 1 H42 8.72

E2.1 1 H141 7.73

E2.1 1 H28 4.76

E2.1 1 H102 8.60

E2.1 1 H74 8.89

E2.1 1 H114 7.74

E2.1 1 H58 7.82

E2.1 1 H128 5.16

E2.1 1 H68 6.59

E2.1 1 H118 6.54

E2.1 1 H01 9.22

E2.1 1 H04 9.02

E2.1 1 H17 10.41

E2.1 1 H10 6.56

E2.1 1 H41 9.03

E2.1 1 H95 7.96

E2.1 1 H76 8.67

E2.1 1 H67 7.90

E2.1 1 H140 6.79

E2.1 1 H57 7.74

E2.1 1 H36 7.79

E2.1 1 H101 7.03

E2.1 1 H79 6.77

E2.1 1 H06 8.89

E2.1 1 H26 5.86

E2.1 1 H39 7.35

E2.1 1 H97 6.88

E2.1 1 H70 7.76

E2.1 1 H13 8.18

E2.1 1 H130 7.25

E2.1 1 H30 7.09

E2.1 1 H22 6.83

E2.1 1 H47 9.44

E2.1 1 H120 6.11

E2.1 1 H16 7.60

E2.1 1 H21 4.65

E2.1 1 H63 7.73

E2.1 1 H33 7.93

E2.1 1 H122 7.16

E2.1 1 H19 9.04

E2.1 1 H143 7.31

E2.1 1 H108 7.13

E2.1 1 H83 6.53

E2.1 1 H124 6.40

E2.1 1 H131 7.68

E2.1 1 H113 7.15

E2.1 1 H02 10.36

E2.1 1 H92 8.85

E2.1 1 H86 10.40

E2.1 1 H109 7.04

E2.1 1 H105 7.99

E2.1 1 H90 9.86

E2.1 1 H53 9.05

E2.1 1 H66 9.29

E2.1 1 H116 9.23

E2.1 1 H127 7.49

E2.1 1 H15 9.55

E2.1 1 H40 7.82

E2.1 1 H51 7.39

E2.1 1 H121 5.55

E2.1 1 H107 8.33

E2.1 1 H69 6.74

E2.1 1 H112 6.89

E2.1 1 H94 7.14

E2.1 1 H20 7.29

E2.1 1 H56 8.03

E2.1 1 H43 9.48

E2.1 1 H129 2.62

E2.1 1 H126 3.81

E2.1 1 H111 5.29

E2.1 1 H62 6.83

E2.1 1 H24 5.86

E2.1 1 H91 7.68

E2.1 1 H89 7.35

E2.1 1 H03 7.36

E2.1 1 H82 7.18

E2.1 1 H12 7.49

E2.1 1 H99 6.78

E2.1 1 H115 8.39

E2.1 1 H119 3.69

E2.1 1 H145 6.14

E2.1 1 H142 5.27

E2.1 1 H135 8.58

E2.1 1 H104 6.70

E2.1 1 H35 8.21

E2.1 1 H59 7.93

E2.1 1 H100 7.88

E2.1 1 H18 9.99

E2.1 1 H32 6.55

E2.1 1 H46 6.81

E2.1 1 H25 6.06

E2.1 1 H05 8.59

E2.1 1 H81 9.35

E2.1 1 H65 6.03

E2.1 1 H78 9.14

E2.1 1 H85 6.59

E2.1 1 H75 7.94

E2.1 1 H29 7.25

E2.1 2 H119 4.78

E2.1 2 H39 10.32

E2.1 2 H92 8.78

E2.1 2 H56 8.53

E2.1 2 H48 9.96

E2.1 2 H66 8.66

E2.1 2 H139 6.57

E2.1 2 H78 8.22

E2.1 2 H105 5.72

E2.1 2 H32 8.10

E2.1 2 H109 6.17

E2.1 2 H40 8.07

E2.1 2 H107 7.96

E2.1 2 H128 4.38

E2.1 2 H27 8.91

E2.1 2 H59 7.95

E2.1 2 H67 7.93

E2.1 2 H83 6.10

E2.1 2 H01 10.72

E2.1 2 H116 7.29

E2.1 2 H75 9.41

E2.1 2 H97 9.24

E2.1 2 H71 8.10

E2.1 2 H144 4.40

E2.1 2 H29 7.60

E2.1 2 H101 8.19

E2.1 2 H96 7.63

E2.1 2 H51 7.81

E2.1 2 H132 9.45

E2.1 2 H35 9.07

E2.1 2 H43 10.34

E2.1 2 H10 8.09

E2.1 2 H88 9.52

E2.1 2 H63 6.04

E2.1 2 H141 8.56

E2.1 2 H124 6.59

E2.1 2 H81 10.18

E2.1 2 H123 7.80

E2.1 2 H112 7.06

E2.1 2 H23 7.47

E2.1 2 H86 9.93

E2.1 2 H53 10.27

E2.1 2 H146 8.18

E2.1 2 H45 9.12

E2.1 2 H104 7.96

E2.1 2 H85 7.57

E2.1 2 H140 7.05

E2.1 2 H26 7.84

E2.1 2 H90 10.78

E2.1 2 H115 7.05

E2.1 2 H126 4.74

E2.1 2 H55 9.71

E2.1 2 H122 5.29

E2.1 2 H74 9.37

E2.1 2 H50 7.38

E2.1 2 H65 7.17

E2.1 2 H69 7.47

E2.1 2 H62 7.64

E2.1 2 H70 7.82

E2.1 2 H143 9.05

E2.1 2 H100 9.50

E2.1 2 H28 5.21

E2.1 2 H131 8.36

E2.1 2 H106 9.14

E2.1 2 H114 7.89

E2.1 2 H82 8.45

E2.1 2 H02 10.21

E2.1 2 H34 9.44

E2.1 2 H80 8.37

E2.1 2 H111 8.21

E2.1 2 H130 9.13

E2.1 2 H31 9.51

E2.1 2 H18 9.94

E2.1 2 H99 8.92

E2.1 2 H42 8.70

E2.1 2 H21 6.39

E2.1 2 H127 7.31

E2.1 2 H95 8.74

E2.1 2 H22 8.21

E2.1 2 H13 8.53

E2.1 2 H47 10.26

E2.1 2 H77 8.23

E2.1 2 H15 8.18

E2.1 2 H118 6.12

E2.1 2 H91 8.99

E2.1 2 H58 9.55

E2.1 2 H20 9.08

E2.1 2 H121 5.84

E2.1 2 H05 10.03

E2.1 2 H102 6.76

E2.1 2 H17 10.19

E2.1 2 H12 7.88

E2.1 2 H113 8.05

E2.1 2 H33 7.36

E2.1 2 H120 5.78

E2.1 2 H19 6.89

E2.1 2 H68 7.51

E2.1 2 H87 5.88

E2.1 2 H93 7.81

E2.1 2 H46 5.95

E2.1 2 H129 3.32

E2.1 2 H04 7.71

E2.1 2 H16 6.93

E2.1 2 H06 8.11

E2.1 2 H64 6.33

E2.1 2 H89 8.06

E2.1 2 H57 7.83

E2.1 2 H84 8.49

E2.1 2 H103 8.10

E2.1 2 H145 7.46

E2.1 2 H76 8.05

E2.1 2 H61 8.40

E2.1 2 H108 7.00

E2.1 2 H125 7.77

E2.1 2 H24 5.84

E2.1 2 H41 7.67

E2.1 2 H79 6.09

E2.1 2 H14 7.84

E2.1 2 H03 10.00

E2.1 2 H135 7.85

E2.1 2 H94 6.16

E2.1 2 H142 5.42

E2.1 2 H25 3.55

E2.1 2 H11 8.67

E2.1 2 H110 5.06

E2.1 2 H98 5.54

E2.1 2 H36 5.08

E2.1 2 H117 4.03

E2.1 2 H30 8.03

E3.1 1 H96 5.98

E3.1 1 H117 6.14

E3.1 1 H34 6.20

E3.1 1 H23 4.32

E3.1 1 H14 6.70

E3.1 1 H123 5.32

E3.1 1 H132 4.14

E3.1 1 H77 7.37

E3.1 1 H106 6.17

E3.1 1 H139 8.24

E3.1 1 H11 6.79

E3.1 1 H87 6.56

E3.1 1 H31 5.74

E3.1 1 H27 6.11

E3.1 1 H64 4.82

E3.1 1 H61 4.77

E3.1 1 H88 6.34

E3.1 1 H93 6.03

E3.1 1 H45 4.94

E3.1 1 H55 6.14

E3.1 1 H146 4.66

E3.1 1 H98 4.28

E3.1 1 H84 5.51

E3.1 1 H125 6.60

E3.1 1 H110 4.71

E3.1 1 H50 6.35

E3.1 1 H144 4.03

E3.1 1 H103 7.00

E3.1 1 H48 4.75

E3.1 1 H71 4.41

E3.1 1 H80 4.86

E3.1 1 H42 4.34

E3.1 1 H141 5.57

E3.1 1 H28 4.56

E3.1 1 H102 6.94

E3.1 1 H74 5.28

E3.1 1 H114 7.30

E3.1 1 H58 5.33

E3.1 1 H128 3.39

E3.1 1 H68 4.24

E3.1 1 H118 5.41

E3.1 1 H01 6.97

E3.1 1 H04 6.60

E3.1 1 H17 6.14

E3.1 1 H10 4.47

E3.1 1 H41 4.12

E3.1 1 H95 5.87

E3.1 1 H76 6.21

E3.1 1 H67 5.64

E3.1 1 H140 4.69

E3.1 1 H57 6.96

E3.1 1 H36 6.06

E3.1 1 H101 5.95

E3.1 1 H79 6.58

E3.1 1 H06 5.95

E3.1 1 H26 3.94

E3.1 1 H39 5.09

E3.1 1 H97 5.00

E3.1 1 H70 5.29

E3.1 1 H13 7.09

E3.1 1 H130 4.55

E3.1 1 H30 6.39

E3.1 1 H22 7.70

E3.1 1 H47 5.34

E3.1 1 H120 2.55

E3.1 1 H16 4.97

E3.1 1 H21 4.41

E3.1 1 H63 5.21

E3.1 1 H33 5.06

E3.1 1 H122 5.10

E3.1 1 H19 6.57

E3.1 1 H143 5.69

E3.1 1 H108 4.96

E3.1 1 H83 4.99

E3.1 1 H124 4.61

E3.1 1 H131 4.83

E3.1 1 H113 4.22

E3.1 1 H02 8.68

E3.1 1 H92 5.26

E3.1 1 H86 7.05

E3.1 1 H109 6.01

E3.1 1 H105 4.75

E3.1 1 H90 4.44

E3.1 1 H53 6.54

E3.1 1 H66 6.87

E3.1 1 H116 6.59

E3.1 1 H127 5.79

E3.1 1 H15 7.82

E3.1 1 H40 4.86

E3.1 1 H51 5.36

E3.1 1 H121 4.68

E3.1 1 H107 4.54

E3.1 1 H69 5.54

E3.1 1 H112 6.54

E3.1 1 H94 5.66

E3.1 1 H20 5.03

E3.1 1 H56 7.40

E3.1 1 H43 7.25

E3.1 1 H129 2.71

E3.1 1 H126 4.58

E3.1 1 H111 6.69

E3.1 1 H62 6.88

E3.1 1 H24 4.74

E3.1 1 H91 7.48

E3.1 1 H89 4.61

E3.1 1 H03 7.44

E3.1 1 H82 6.40

E3.1 1 H12 7.51

E3.1 1 H99 7.36

E3.1 1 H115 7.87

E3.1 1 H119 3.55

E3.1 1 H145 6.14

E3.1 1 H142 6.78

E3.1 1 H135 6.42

E3.1 1 H104 7.12

E3.1 1 H35 7.49

E3.1 1 H59 6.22

E3.1 1 H100 6.51

E3.1 1 H18 8.35

E3.1 1 H32 5.87

E3.1 1 H46 6.83

E3.1 1 H25 5.25

E3.1 1 H05 6.59

E3.1 1 H81 6.84

E3.1 1 H65 5.63

E3.1 1 H78 5.98

E3.1 1 H85 5.02

E3.1 1 H75 5.12

E3.1 1 H29 5.76

E3.1 2 H119 4.76

E3.1 2 H39 7.47

E3.1 2 H92 6.01

E3.1 2 H56 6.20

E3.1 2 H48 5.52

E3.1 2 H66 6.37

E3.1 2 H139 6.39

E3.1 2 H78 6.16

E3.1 2 H105 6.90

E3.1 2 H32 5.94

E3.1 2 H109 5.66

E3.1 2 H40 5.03

E3.1 2 H107 7.08

E3.1 2 H128 3.78

E3.1 2 H27 7.64

E3.1 2 H59 6.61

E3.1 2 H67 6.32

E3.1 2 H83 3.68

E3.1 2 H01 8.94

E3.1 2 H116 6.40

E3.1 2 H75 4.32

E3.1 2 H97 5.48

E3.1 2 H71 5.39

E3.1 2 H144 4.75

E3.1 2 H29 5.47

E3.1 2 H101 5.89

E3.1 2 H96 7.16

E3.1 2 H51 4.84

E3.1 2 H132 3.97

E3.1 2 H35 6.17

E3.1 2 H43 5.14

E3.1 2 H10 4.05

E3.1 2 H88 6.91

E3.1 2 H63 7.03

E3.1 2 H141 6.27

E3.1 2 H124 3.44

E3.1 2 H81 5.15

E3.1 2 H123 6.66

E3.1 2 H112 4.99

E3.1 2 H23 5.07

E3.1 2 H86 5.50

E3.1 2 H53 6.45

E3.1 2 H146 4.37

E3.1 2 H45 4.53

E3.1 2 H104 5.06

E3.1 2 H85 3.81

E3.1 2 H140 4.33

E3.1 2 H26 5.49

E3.1 2 H90 5.79

E3.1 2 H115 4.46

E3.1 2 H126 4.07

E3.1 2 H55 4.12

E3.1 2 H122 3.40

E3.1 2 H74 4.49

E3.1 2 H50 2.31

E3.1 2 H65 3.98

E3.1 2 H69 4.32

E3.1 2 H62 3.51

E3.1 2 H70 3.13

E3.1 2 H143 4.69

E3.1 2 H100 3.49

E3.1 2 H28 3.51

E3.1 2 H131 5.01

E3.1 2 H106 3.95

E3.1 2 H114 5.26

E3.1 2 H82 4.06

E3.1 2 H02 8.95

E3.1 2 H34 5.07

E3.1 2 H80 2.62

E3.1 2 H111 5.65

E3.1 2 H130 5.85

E3.1 2 H31 4.51

E3.1 2 H18 6.60

E3.1 2 H99 5.41

E3.1 2 H42 4.62

E3.1 2 H21 4.52

E3.1 2 H127 5.26

E3.1 2 H95 6.18

E3.1 2 H22 4.56

E3.1 2 H13 6.06

E3.1 2 H47 5.70

E3.1 2 H77 5.65

E3.1 2 H15 5.38

E3.1 2 H118 5.91

E3.1 2 H91 4.58

E3.1 2 H58 5.91

E3.1 2 H20 5.89

E3.1 2 H121 6.02

E3.1 2 H05 6.42

E3.1 2 H102 7.42

E3.1 2 H17 6.30

E3.1 2 H12 5.95

E3.1 2 H113 8.89

E3.1 2 H33 7.66

E3.1 2 H120 7.52

E3.1 2 H19 7.64

E3.1 2 H68 6.48

E3.1 2 H87 7.48

E3.1 2 H93 7.04

E3.1 2 H46 7.31

E3.1 2 H129 3.27

E3.1 2 H04 9.50

E3.1 2 H16 6.57

E3.1 2 H06 6.01

E3.1 2 H64 5.79

E3.1 2 H89 5.62

E3.1 2 H57 7.81

E3.1 2 H84 6.21

E3.1 2 H103 6.17

E3.1 2 H145 6.82

E3.1 2 H76 6.89

E3.1 2 H61 7.13

E3.1 2 H108 6.40

E3.1 2 H125 7.74

E3.1 2 H24 6.10

E3.1 2 H41 4.87

E3.1 2 H79 5.70

E3.1 2 H14 6.75

E3.1 2 H03 7.54

E3.1 2 H135 6.90

E3.1 2 H94 6.76

E3.1 2 H142 7.17

E3.1 2 H25 2.77

E3.1 2 H11 7.28

E3.1 2 H110 4.52

E3.1 2 H98 6.21

E3.1 2 H36 6.02

E3.1 2 H117 6.00

E3.1 2 H30 5.44

E1.2 1 H12 11.99

E1.2 1 H01 14.28

E1.2 1 H02 9.91

E1.2 1 H03 14.24

E1.2 1 H13 11.23

E1.2 1 H14 12.16

E1.2 1 H15 11.66

E1.2 1 H16 9.74

E1.2 1 H17 11.95

E1.2 1 H18 10.45

E1.2 1 H04 11.52

E1.2 1 H19 10.38

E1.2 1 H05 10.33

E1.2 1 H06 12.30

E1.2 1 H11 12.99

E1.2 2 H02 13.18

E1.2 2 H17 13.91

E1.2 2 H13 12.82

E1.2 2 H19 13.56

E1.2 2 H18 13.03

E1.2 2 H14 13.38

E1.2 2 H06 13.99

E1.2 2 H11 13.45

E1.2 2 H15 14.54

E1.2 2 H05 13.56

E1.2 2 H04 12.84

E1.2 2 H03 14.91

E1.2 2 H16 13.05

E1.2 2 H12 11.98

E1.2 2 H01 12.84

E1.2 3 H11 13.30

E1.2 3 H12 16.43

E1.2 3 H01 13.29

E1.2 3 H05 13.86

E1.2 3 H14 14.53

E1.2 3 H02 16.51

E1.2 3 H17 14.24

E1.2 3 H19 14.59

E1.2 3 H03 15.78

E1.2 3 H06 13.62

E1.2 3 H16 11.64

E1.2 3 H13 14.09

E1.2 3 H04 14.88

E1.2 3 H15 11.84

E1.2 3 H18 13.97

E3.2 1 H12 9.14

E3.2 1 H01 10.64

E3.2 1 H02 11.23

E3.2 1 H03 8.61

E3.2 1 H13 9.07

E3.2 1 H14 8.07

E3.2 1 H15 7.66

E3.2 1 H16 9.79

E3.2 1 H17 8.68

E3.2 1 H18 9.18

E3.2 1 H04 9.35

E3.2 1 H19 11.55

E3.2 1 H05 10.04

E3.2 1 H06 10.75

E3.2 1 H11 9.70

E3.2 2 H02 8.96

E3.2 2 H17 10.73

E3.2 2 H13 8.71

E3.2 2 H19 10.18

E3.2 2 H18 8.66

E3.2 2 H14 6.98

E3.2 2 H06 9.72

E3.2 2 H11 9.86

E3.2 2 H15 6.29

E3.2 2 H05 10.11

E3.2 2 H04 7.74

E3.2 2 H03 7.85

E3.2 2 H16 7.39

E3.2 2 H12 8.19

E3.2 2 H01 7.89

E3.2 3 H11 9.96

E3.2 3 H12 9.82

E3.2 3 H01 9.88

E3.2 3 H05 8.23

E3.2 3 H14 5.08

E3.2 3 H02 8.13

E3.2 3 H17 10.24

E3.2 3 H19 9.97

E3.2 3 H03 7.72

E3.2 3 H06 9.24

E3.2 3 H16 9.11

E3.2 3 H13 8.22

E3.2 3 H04 8.95

E3.2 3 H15 7.14

E3.2 3 H18 8.27

E2.2 1 H12 13.98

E2.2 1 H01 16.84

E2.2 1 H02 17.50

E2.2 1 H03 18.68

E2.2 1 H13 15.10

E2.2 1 H14 15.99

E2.2 1 H15 15.82

E2.2 1 H16 14.79

E2.2 1 H17 18.39

E2.2 1 H18 16.66

E2.2 1 H04 12.71

E2.2 1 H19 19.21

E2.2 1 H05 19.19

E2.2 1 H06 12.64

E2.2 1 H11 18.27

E2.2 2 H02 17.82

E2.2 2 H17 15.52

E2.2 2 H13 15.76

E2.2 2 H19 13.70

E2.2 2 H18 18.58

E2.2 2 H14 17.50

E2.2 2 H06 15.53

E2.2 2 H11 16.41

E2.2 2 H15 16.63

E2.2 2 H05 16.71

E2.2 2 H04 18.49

E2.2 2 H03 20.38

E2.2 2 H16 14.35

E2.2 2 H12 15.96

E2.2 2 H01 18.38

E2.2 3 H11 14.26

E2.2 3 H12 16.25

E2.2 3 H01 20.45

E2.2 3 H05 17.18

E2.2 3 H14 16.09

E2.2 3 H02 21.76

E2.2 3 H17 16.74

E2.2 3 H19 15.33

E2.2 3 H03 21.83

E2.2 3 H06 17.72

E2.2 3 H16 14.14

E2.2 3 H13 15.49

E2.2 3 H04 17.60

E2.2 3 H15 16.94

E2.2 3 H18 15.49

E4.2 1 H12 14.24

E4.2 1 H01 14.15

E4.2 1 H02 11.24

E4.2 1 H03 11.67

E4.2 1 H13 10.96

E4.2 1 H14 12.31

E4.2 1 H15 11.31

E4.2 1 H16 10.16

E4.2 1 H17 10.57

E4.2 1 H18 10.74

E4.2 1 H04 10.77

E4.2 1 H19 8.91

E4.2 1 H05 10.54

E4.2 1 H06 10.88

E4.2 1 H11 10.43

E4.2 2 H02 16.60

E4.2 2 H17 11.66

E4.2 2 H13 10.97

E4.2 2 H19 10.23

E4.2 2 H18 12.45

E4.2 2 H14 10.31

E4.2 2 H06 12.98

E4.2 2 H11 11.98

E4.2 2 H15 11.04

E4.2 2 H05 10.29

E4.2 2 H04 10.48

E4.2 2 H03 10.92

E4.2 2 H16 11.61

E4.2 2 H12 10.99

E4.2 2 H01 11.23

E4.2 3 H11 12.79

E4.2 3 H12 12.02

E4.2 3 H01 14.65

E4.2 3 H05 12.68

E4.2 3 H14 12.48

E4.2 3 H02 11.74

E4.2 3 H17 9.70

E4.2 3 H19 10.23

E4.2 3 H03 11.66

E4.2 3 H06 11.12

E4.2 3 H16 7.74

E4.2 3 H13 10.35

E4.2 3 H04 11.28

E4.2 3 H15 9.14

E4.2 3 H18 9.19

E1.3 1 H01 14.45

E1.3 1 H02 14.16

E1.3 1 H03 15.57

E1.3 1 H04 14.56

E1.3 1 H05 15.23

E1.3 1 H06 13.49

E1.3 1 H07 12.52

E1.3 1 H08 14.52

E1.3 1 H09 11.05

E1.3 1 H10 11.49

E1.3 1 H11 12.97

E1.3 2 H06 13.20

E1.3 2 H02 13.96

E1.3 2 H09 13.74

E1.3 2 H10 15.06

E1.3 2 H04 16.04

E1.3 2 H07 12.72

E1.3 2 H03 13.03

E1.3 2 H08 17.67

E1.3 2 H01 15.23

E1.3 2 H05 13.09

E1.3 2 H11 14.46

E1.3 3 H09 13.41

E1.3 3 H04 13.75

E1.3 3 H06 14.93

E1.3 3 H11 15.03

E1.3 3 H03 15.54

E1.3 3 H02 15.19

E1.3 3 H07 15.90

E1.3 3 H05 14.06

E1.3 3 H10 15.86

E1.3 3 H08 15.46

E1.3 3 H01 17.26

E1.3 4 H07 16.42

E1.3 4 H05 16.87

E1.3 4 H09 15.73

E1.3 4 H06 16.64

E1.3 4 H02 15.21

E1.3 4 H08 16.95

E1.3 4 H03 12.01

E1.3 4 H04 16.35

E1.3 4 H10 15.54

E1.3 4 H11 15.17

E1.3 4 H01 15.29

E1.4 1 H01 15.83

E1.4 1 H02 17.38

E1.4 1 H03 21.87

E1.4 1 H04 16.81

E1.4 1 H05 21.20

E1.4 1 H06 16.06

E1.4 1 H07 19.59

E1.4 1 H08 13.56

E1.4 1 H09 13.45

E1.4 1 H10 15.09

E1.4 1 H11 13.35

E1.4 2 H06 14.80

E1.4 2 H02 15.94

E1.4 2 H09 17.86

E1.4 2 H10 15.44

E1.4 2 H04 14.70

E1.4 2 H07 18.39

E1.4 2 H03 19.13

E1.4 2 H08 13.46

E1.4 2 H01 14.76

E1.4 2 H05 19.23

E1.4 2 H11 16.51

E1.4 3 H09 16.73

E1.4 3 H04 16.07

E1.4 3 H06 12.79

E1.4 3 H11 17.88

E1.4 3 H03 21.22

E1.4 3 H02 16.47

E1.4 3 H07 14.56

E1.4 3 H05 15.53

E1.4 3 H10 13.20

E1.4 3 H08 16.45

E1.4 3 H01 12.48

E1.4 4 H07 17.37

E1.4 4 H05 20.14

E1.4 4 H09 17.77

E1.4 4 H06 17.35

E1.4 4 H02 17.50

E1.4 4 H08 17.06

E1.4 4 H03 16.51

E1.4 4 H04 12.47

E1.4 4 H10 12.28

E1.4 4 H11 16.28

E1.4 4 H01 13.65

E2.3 1 H01 10.97

E2.3 1 H02 14.38

E2.3 1 H03 15.98

E2.3 1 H04 11.61

E2.3 1 H05 6.45

E2.3 1 H06 5.33

E2.3 1 H07 9.36

E2.3 1 H08 7.19

E2.3 1 H09 7.66

E2.3 1 H10 6.61

E2.3 1 H11 6.80

E2.3 2 H06 10.00

E2.3 2 H02 16.11

E2.3 2 H09 7.88

E2.3 2 H10 8.79

E2.3 2 H04 10.87

E2.3 2 H07 13.74

E2.3 2 H03 11.70

E2.3 2 H08 6.36

E2.3 2 H01 7.41

E2.3 2 H05 5.05

E2.3 2 H11 6.07

E2.3 3 H09 7.01

E2.3 3 H04 13.84

E2.3 3 H06 11.82

E2.3 3 H11 10.58

E2.3 3 H03 10.59

E2.3 3 H02 12.08

E2.3 3 H07 12.39

E2.3 3 H05 7.36

E2.3 3 H10 5.90

E2.3 3 H08 9.97

E2.3 3 H01 9.15

E2.3 4 H07 10.02

E2.3 4 H05 10.01

E2.3 4 H09 6.45

E2.3 4 H06 11.61

E2.3 4 H02 12.24

E2.3 4 H08 9.33

E2.3 4 H03 10.86

E2.3 4 H04 12.87

E2.3 4 H10 6.06

E2.3 4 H11 8.90

E2.3 4 H01 10.21

E2.4 1 H01 13.04

E2.4 1 H02 10.79

E2.4 1 H03 13.05

E2.4 1 H04 11.61

E2.4 1 H05 10.92

E2.4 1 H06 11.56

E2.4 1 H07 14.28

E2.4 1 H08 10.81

E2.4 1 H09 7.24

E2.4 1 H10 8.50

E2.4 1 H11 10.77

E2.4 2 H06 12.99

E2.4 2 H02 14.00

E2.4 2 H09 9.52

E2.4 2 H10 11.20

E2.4 2 H04 13.53

E2.4 2 H07 14.18

E2.4 2 H03 12.02

E2.4 2 H08 13.68

E2.4 2 H01 14.34

E2.4 2 H05 15.08

E2.4 2 H11 13.62

E2.4 3 H09 10.63

E2.4 3 H04 14.96

E2.4 3 H06 12.36

E2.4 3 H11 13.72

E2.4 3 H03 13.99

E2.4 3 H02 14.13

E2.4 3 H07 14.80

E2.4 3 H05 15.67

E2.4 3 H10 9.52

E2.4 3 H08 13.50

E2.4 3 H01 12.80

E2.4 4 H07 13.18

E2.4 4 H05 13.41

E2.4 4 H09 11.20

E2.4 4 H06 14.23

E2.4 4 H02 15.03

E2.4 4 H08 14.89

E2.4 4 H03 14.51

E2.4 4 H04 13.38

E2.4 4 H10 11.31

E2.4 4 H11 13.08

E2.4 4 H01 13.30

E4.3 1 H01 17.98

E4.3 1 H02 17.60

E4.3 1 H03 20.94

E4.3 1 H04 17.34

E4.3 1 H05 15.79

E4.3 1 H06 17.48

E4.3 1 H07 16.96

E4.3 1 H08 13.78

E4.3 1 H09 16.88

E4.3 1 H10 11.57

E4.3 1 H11 13.83

E4.3 2 H06 19.42

E4.3 2 H02 19.54

E4.3 2 H09 17.55

E4.3 2 H10 17.37

E4.3 2 H04 16.16

E4.3 2 H07 18.89

E4.3 2 H03 19.13

E4.3 2 H08 16.32

E4.3 2 H01 18.39

E4.3 2 H05 14.88

E4.3 2 H11 14.85

E4.3 3 H09 17.01

E4.3 3 H04 16.01

E4.3 3 H06 18.41

E4.3 3 H11 15.80

E4.3 3 H03 17.47

E4.3 3 H02 18.45

E4.3 3 H07 17.22

E4.3 3 H05 16.53

E4.3 3 H10 14.43

E4.3 3 H08 13.86

E4.3 3 H01 16.37

E4.3 4 H07 17.90

E4.3 4 H05 18.65

E4.3 4 H09 19.65

E4.3 4 H06 17.61

E4.3 4 H02 16.34

E4.3 4 H08 15.93

E4.3 4 H03 17.96

E4.3 4 H04 15.32

E4.3 4 H10 12.84

E4.3 4 H11 14.42

E4.3 4 H01 16.64

E4.4 1 H01 19.38

E4.4 1 H02 17.45

E4.4 1 H03 19.72

E4.4 1 H04 15.76

E4.4 1 H05 16.02

E4.4 1 H06 16.19

E4.4 1 H07 11.81

E4.4 1 H08 17.91

E4.4 1 H09 15.41

E4.4 1 H10 12.22

E4.4 1 H11 13.49

E4.4 2 H06 14.79

E4.4 2 H02 18.80

E4.4 2 H09 16.03

E4.4 2 H10 15.22

E4.4 2 H04 18.37

E4.4 2 H07 14.02

E4.4 2 H03 19.29

E4.4 2 H08 19.38

E4.4 2 H01 20.28

E4.4 2 H05 15.87

E4.4 2 H11 15.05

E4.4 3 H09 12.59

E4.4 3 H04 16.26

E4.4 3 H06 18.92

E4.4 3 H11 18.11

E4.4 3 H03 17.93

E4.4 3 H02 15.38

E4.4 3 H07 11.88

E4.4 3 H05 16.74

E4.4 3 H10 11.84

E4.4 3 H08 15.87

E4.4 3 H01 17.63

E4.4 4 H07 14.29

E4.4 4 H05 17.41

E4.4 4 H09 18.75

E4.4 4 H06 15.44

E4.4 4 H02 17.98

E4.4 4 H08 18.05

E4.4 4 H03 17.60

E4.4 4 H04 18.28

E4.4 4 H10 14.34

E4.4 4 H11 15.25

E4.4 4 H01 13.92

E5.3 1 H01 12.79

E5.3 1 H02 13.01

E5.3 1 H03 12.16

E5.3 1 H04 11.59

E5.3 1 H05 10.09

E5.3 1 H06 8.26

E5.3 1 H07 9.00

E5.3 1 H08 11.01

E5.3 1 H09 8.13

E5.3 1 H10 10.38

E5.3 1 H11 14.92

E5.3 2 H06 8.28

E5.3 2 H02 12.57

E5.3 2 H09 9.94

E5.3 2 H10 10.27

E5.3 2 H04 8.68

E5.3 2 H07 8.55

E5.3 2 H03 10.89

E5.3 2 H08 1.05

E5.3 2 H01 12.10

E5.3 2 H05 7.63

E5.3 2 H11 12.34

E5.3 3 H09 10.34

E5.3 3 H04 8.99

E5.3 3 H06 8.27

E5.3 3 H11 14.62

E5.3 3 H03 11.44

E5.3 3 H02 13.12

E5.3 3 H07 10.99

E5.3 3 H05 8.24

E5.3 3 H10 9.93

E5.3 3 H08 12.33

E5.3 3 H01 13.13

E5.3 4 H07 10.01

E5.3 4 H05 8.38

E5.3 4 H09 8.98

E5.3 4 H06 6.96

E5.3 4 H02 11.83

E5.3 4 H08 12.00

E5.3 4 H03 12.48

E5.3 4 H04 9.67

E5.3 4 H10 10.13

E5.3 4 H11 11.60

E5.3 4 H01 13.36

E5.4 1 H01 8.85

E5.4 1 H02 11.10

E5.4 1 H03 13.35

E5.4 1 H04 11.57

E5.4 1 H05 11.22

E5.4 1 H06 11.96

E5.4 1 H07 11.27

E5.4 1 H08 11.08

E5.4 1 H09 8.69

E5.4 1 H10 8.41

E5.4 1 H11 8.69

E5.4 2 H06 10.13

E5.4 2 H02 13.48

E5.4 2 H09 9.84

E5.4 2 H10 12.25

E5.4 2 H04 14.31

E5.4 2 H07 10.66

E5.4 2 H03 14.28

E5.4 2 H08 12.72

E5.4 2 H01 13.38

E5.4 2 H05 9.30

E5.4 2 H11 11.75

E5.4 3 H09 10.24

E5.4 3 H04 14.13

E5.4 3 H06 9.24

E5.4 3 H11 13.40

E5.4 3 H03 14.72

E5.4 3 H02 15.01

E5.4 3 H07 12.12

E5.4 3 H05 14.10

E5.4 3 H10 13.54

E5.4 3 H08 12.55

E5.4 3 H01 15.38

E5.4 4 H07 8.28

E5.4 4 H05 9.19

E5.4 4 H09 10.74

E5.4 4 H06 13.54

E5.4 4 H02 13.21

E5.4 4 H08 13.69

E5.4 4 H03 14.92

E5.4 4 H04 13.64

E5.4 4 H10 9.26

E5.4 4 H11 11.27

E5.4 4 H01 12.94

E3.3 1 H01 7.41

E3.3 1 H02 10.15

E3.3 1 H03 11.70

E3.3 1 H04 11.60

E3.3 1 H05 9.19

E3.3 1 H06 11.48

E3.3 1 H07 12.73

E3.3 1 H08 5.27

E3.3 1 H09 7.07

E3.3 1 H10 7.18

E3.3 1 H11 12.98

E3.3 2 H06 6.97

E3.3 2 H02 12.90

E3.3 2 H09 10.54

E3.3 2 H10 8.24

E3.3 2 H04 12.03

E3.3 2 H07 14.77

E3.3 2 H03 10.92

E3.3 2 H08 8.55

E3.3 2 H01 9.82

E3.3 2 H05 9.26

E3.3 2 H11 17.83

E3.3 3 H09 8.89

E3.3 3 H04 16.89

E3.3 3 H06 12.07

E3.3 3 H11 15.35

E3.3 3 H03 12.91

E3.3 3 H02 13.71

E3.3 3 H07 15.64

E3.3 3 H05 9.47

E3.3 3 H10 7.19

E3.3 3 H08 11.47

E3.3 3 H01 12.09

E3.3 4 H07 15.77

E3.3 4 H05 10.61

E3.3 4 H09 9.86

E3.3 4 H06 10.32

E3.3 4 H02 12.71

E3.3 4 H08 8.16

E3.3 4 H03 12.21

E3.3 4 H04 9.75

E3.3 4 H10 6.83

E3.3 4 H11 14.23

E3.3 4 H01 15.89

E3.4 1 H01 13.58

E3.4 1 H02 19.57

E3.4 1 H03 26.44

E3.4 1 H04 16.74

E3.4 1 H05 13.09

E3.4 1 H06 14.48

E3.4 1 H07 16.32

E3.4 1 H08 16.15

E3.4 1 H09 11.64

E3.4 1 H10 18.16

E3.4 1 H11 18.75

E3.4 2 H06 19.07

E3.4 2 H02 25.17

E3.4 2 H09 17.05

E3.4 2 H10 14.45

E3.4 2 H04 18.82

E3.4 2 H07 17.03

E3.4 2 H03 17.79

E3.4 2 H08 19.58

E3.4 2 H01 13.45

E3.4 2 H05 16.54

E3.4 2 H11 21.36

E3.4 3 H09 22.12

E3.4 3 H04 15.61

E3.4 3 H06 15.37

E3.4 3 H11 18.76

E3.4 3 H03 23.33

E3.4 3 H02 15.77

E3.4 3 H07 24.87

E3.4 3 H05 13.50

E3.4 3 H10 18.23

E3.4 3 H08 11.96

E3.4 3 H01 26.41

E3.4 4 H07 22.88

E3.4 4 H05 17.91

E3.4 4 H09 16.60

E3.4 4 H06 18.03

E3.4 4 H02 25.19

E3.4 4 H08 24.57

E3.4 4 H03 25.09

E3.4 4 H04 21.80

E3.4 4 H10 18.96

E3.4 4 H11 23.20

E3.4 4 H01 17.27

E6.3 1 H01 13.32

E6.3 1 H02 10.86

E6.3 1 H03 12.00

E6.3 1 H04 10.85

E6.3 1 H05 14.37

E6.3 1 H06 11.64

E6.3 1 H07 12.89

E6.3 1 H08 9.07

E6.3 1 H09 15.77

E6.3 1 H10 12.51

E6.3 1 H11 9.85

E6.3 2 H06 8.11

E6.3 2 H02 8.96

E6.3 2 H09 13.29

E6.3 2 H10 11.79

E6.3 2 H04 10.20

E6.3 2 H07 11.01

E6.3 2 H03 12.08

E6.3 2 H08 10.39

E6.3 2 H01 9.04

E6.3 2 H05 9.51

E6.3 2 H11 11.22

E6.3 3 H09 12.35

E6.3 3 H04 8.90

E6.3 3 H06 11.78

E6.3 3 H11 8.60

E6.3 3 H03 11.35

E6.3 3 H02 9.34

E6.3 3 H07 9.63

E6.3 3 H05 7.89

E6.3 3 H10 11.01

E6.3 3 H08 9.98

E6.3 3 H01 9.03

E6.3 4 H07 9.21

E6.3 4 H05 11.67

E6.3 4 H09 9.75

E6.3 4 H06 8.96

E6.3 4 H02 14.73

E6.3 4 H08 7.71

E6.3 4 H03 12.25

E6.3 4 H04 8.82

E6.3 4 H10 9.34

E6.3 4 H11 9.47

E6.3 4 H01 9.76

E6.4 1 H01 15.99

E6.4 1 H02 14.24

E6.4 1 H03 15.63

E6.4 1 H04 11.49

E6.4 1 H05 13.62

E6.4 1 H06 7.97

E6.4 1 H07 16.78

E6.4 1 H08 12.62

E6.4 1 H09 14.31

E6.4 1 H10 10.73

E6.4 1 H11 8.64

E6.4 2 H06 13.72

E6.4 2 H02 13.15

E6.4 2 H09 15.40

E6.4 2 H10 12.93

E6.4 2 H04 15.25

E6.4 2 H07 16.07

E6.4 2 H03 15.17

E6.4 2 H08 12.66

E6.4 2 H01 12.03

E6.4 2 H05 9.88

E6.4 2 H11 7.57

E6.4 3 H09 12.72

E6.4 3 H04 11.50

E6.4 3 H06 14.81

E6.4 3 H11 11.44

E6.4 3 H03 16.39

E6.4 3 H02 10.95

E6.4 3 H07 13.29

E6.4 3 H05 14.79

E6.4 3 H10 13.03

E6.4 3 H08 7.57

E6.4 3 H01 7.45

E6.4 4 H07 13.75

E6.4 4 H05 10.44

E6.4 4 H09 11.84

E6.4 4 H06 10.79

E6.4 4 H02 15.53

E6.4 4 H08 12.83

E6.4 4 H03 15.33

E6.4 4 H04 13.52

E6.4 4 H10 11.09

E6.4 4 H11 11.35

E6.4 4 H01 6.16

E7.3 1 H01 14.09

E7.3 1 H02 18.74

E7.3 1 H03 15.84

E7.3 1 H04 16.58

E7.3 1 H05 14.83

E7.3 1 H06 15.18

E7.3 1 H07 13.71

E7.3 1 H08 13.57

E7.3 1 H09 17.64

E7.3 1 H10 13.36

E7.3 1 H11 13.40

E7.3 2 H06 15.12

E7.3 2 H02 18.57

E7.3 2 H09 17.00

E7.3 2 H10 15.18

E7.3 2 H04 16.15

E7.3 2 H07 14.39

E7.3 2 H03 15.83

E7.3 2 H08 14.00

E7.3 2 H01 15.49

E7.3 2 H05 15.99

E7.3 2 H11 14.88

E7.3 3 H09 16.93

E7.3 3 H04 18.11

E7.3 3 H06 16.95

E7.3 3 H11 15.16

E7.3 3 H03 17.32

E7.3 3 H02 20.18

E7.3 3 H07 16.11

E7.3 3 H05 17.58

E7.3 3 H10 15.49

E7.3 3 H08 15.16

E7.3 3 H01 15.76

E7.3 4 H07 15.49

E7.3 4 H05 15.89

E7.3 4 H09 17.26

E7.3 4 H06 17.33

E7.3 4 H02 20.26

E7.3 4 H08 14.62

E7.3 4 H03 18.77

E7.3 4 H04 18.61

E7.3 4 H10 14.82

E7.3 4 H11 14.94

E7.3 4 H01 13.92

E7.4 1 H01 14.95

E7.4 1 H02 17.19

E7.4 1 H03 17.69

E7.4 1 H04 18.51

E7.4 1 H05 17.41

E7.4 1 H06 18.46

E7.4 1 H07 18.07

E7.4 1 H08 19.03

E7.4 1 H09 16.09

E7.4 1 H10 13.18

E7.4 1 H11 15.06

E7.4 2 H06 16.49

E7.4 2 H02 17.92

E7.4 2 H09 15.38

E7.4 2 H10 16.24

E7.4 2 H04 18.54

E7.4 2 H07 18.63

E7.4 2 H03 19.29

E7.4 2 H08 18.53

E7.4 2 H01 16.78

E7.4 2 H05 18.54

E7.4 2 H11 15.68

E7.4 3 H09 15.37

E7.4 3 H04 17.01

E7.4 3 H06 15.02

E7.4 3 H11 13.43

E7.4 3 H03 14.70

E7.4 3 H02 12.97

E7.4 3 H07 15.81

E7.4 3 H05 18.47

E7.4 3 H10 13.78

E7.4 3 H08 17.82

E7.4 3 H01 15.29

E7.4 4 H07 13.64

E7.4 4 H05 15.37

E7.4 4 H09 15.96

E7.4 4 H06 15.53

E7.4 4 H02 15.45

E7.4 4 H08 15.75

E7.4 4 H03 15.11

E7.4 4 H04 16.77

E7.4 4 H10 14.46

E7.4 4 H11 13.20

E7.4 4 H01 14.65

E8.3 1 H01 5.23

E8.3 1 H02 6.69

E8.3 1 H03 7.27

E8.3 1 H04 7.02

E8.3 1 H05 4.66

E8.3 1 H06 4.69

E8.3 1 H07 7.00

E8.3 1 H08 6.07

E8.3 1 H09 4.07

E8.3 1 H10 5.69

E8.3 1 H11 6.69

E8.3 2 H06 5.62

E8.3 2 H02 6.24

E8.3 2 H09 5.34

E8.3 2 H10 4.51

E8.3 2 H04 5.16

E8.3 2 H07 6.12

E8.3 2 H03 6.17

E8.3 2 H08 5.55

E8.3 2 H01 6.76

E8.3 2 H05 5.31

E8.3 2 H11 5.20

E8.3 3 H09 4.82

E8.3 3 H04 7.36

E8.3 3 H06 5.94

E8.3 3 H11 5.87

E8.3 3 H03 5.83

E8.3 3 H02 5.75

E8.3 3 H07 6.62

E8.3 3 H05 5.43

E8.3 3 H10 5.74

E8.3 3 H08 5.02

E8.3 3 H01 6.11

E8.3 4 H07 6.45

E8.3 4 H05 5.08

E8.3 4 H09 4.74

E8.3 4 H06 5.18

E8.3 4 H02 4.88

E8.3 4 H08 4.80

E8.3 4 H03 6.85

E8.3 4 H04 8.55

E8.3 4 H10 6.42

E8.3 4 H11 6.39

E8.3 4 H01 6.46

E8.4 1 H01 5.76

E8.4 1 H02 5.92

E8.4 1 H03 6.22

E8.4 1 H04 7.87

E8.4 1 H05 4.84

E8.4 1 H06 4.68

E8.4 1 H07 7.28

E8.4 1 H08 5.61

E8.4 1 H09 4.56

E8.4 1 H10 4.54

E8.4 1 H11 4.51

E8.4 2 H06 2.49

E8.4 2 H02 4.65

E8.4 2 H09 3.08

E8.4 2 H10 4.05

E8.4 2 H04 15.58

E8.4 2 H07 5.88

E8.4 2 H03 6.89

E8.4 2 H08 5.53

E8.4 2 H01 7.34

E8.4 2 H05 4.32

E8.4 2 H11 4.96

E8.4 3 H09 3.84

E8.4 3 H04 4.37

E8.4 3 H06 4.43

E8.4 3 H11 4.88

E8.4 3 H03 3.65

E8.4 3 H02 4.91

E8.4 3 H07 6.43

E8.4 3 H05 6.22

E8.4 3 H10 5.47

E8.4 3 H08 6.94

E8.4 3 H01 5.83

E8.4 4 H07 6.80

E8.4 4 H05 3.79

E8.4 4 H09 4.39

E8.4 4 H06 6.09

E8.4 4 H02 4.97

E8.4 4 H08 5.82

E8.4 4 H03 6.59

E8.4 4 H04 6.39

E8.4 4 H10 3.39

E8.4 4 H11 5.19

E8.4 4 H01 7.67

E9.3 1 H01 9.22

E9.3 1 H02 9.48

E9.3 1 H03 9.79

E9.3 1 H04 8.07

E9.3 1 H05 10.10

E9.3 1 H06 8.26

E9.3 1 H07 10.54

E9.3 1 H08 8.78

E9.3 1 H09 10.50

E9.3 1 H10 10.11

E9.3 1 H11 8.57

E9.3 2 H06 8.68

E9.3 2 H02 8.86

E9.3 2 H09 10.36

E9.3 2 H10 9.32

E9.3 2 H04 6.28

E9.3 2 H07 10.85

E9.3 2 H03 7.97

E9.3 2 H08 6.61

E9.3 2 H01 6.83

E9.3 2 H05 9.05

E9.3 2 H11 10.43

E9.3 3 H09 13.91

E9.3 3 H04 10.24

E9.3 3 H06 10.22

E9.3 3 H11 12.72

E9.3 3 H03 14.79

E9.3 3 H02 12.08

E9.3 3 H07 13.85

E9.3 3 H05 12.51

E9.3 3 H10 9.72

E9.3 3 H08 11.60

E9.3 3 H01 11.85

E9.3 4 H07 11.90

E9.3 4 H05 12.17

E9.3 4 H09 12.65

E9.3 4 H06 11.53

E9.3 4 H02 12.83

E9.3 4 H08 12.99

E9.3 4 H03 12.49

E9.3 4 H04 11.15

E9.3 4 H10 8.51

E9.3 4 H11 10.03

E9.3 4 H01 13.95

E9.4 1 H01 16.45

E9.4 1 H02 16.22

E9.4 1 H03 18.72

E9.4 1 H04 17.13

E9.4 1 H05 19.22

E9.4 1 H06 15.90

E9.4 1 H07 13.83

E9.4 1 H08 16.10

E9.4 1 H09 15.62

E9.4 1 H10 14.92

E9.4 1 H11 14.74

E9.4 2 H06 12.68

E9.4 2 H02 16.02

E9.4 2 H09 16.66

E9.4 2 H10 15.01

E9.4 2 H04 14.85

E9.4 2 H07 14.86

E9.4 2 H03 16.44

E9.4 2 H08 17.53

E9.4 2 H01 17.96

E9.4 2 H05 14.88

E9.4 2 H11 15.38

E9.4 3 H09 14.43

E9.4 3 H04 16.47

E9.4 3 H06 15.21

E9.4 3 H11 14.73

E9.4 3 H03 16.93

E9.4 3 H02 16.84

E9.4 3 H07 15.51

E9.4 3 H05 15.04

E9.4 3 H10 16.75

E9.4 3 H08 13.10

E9.4 3 H01 14.79

E9.4 4 H07 20.39

E9.4 4 H05 13.42

E9.4 4 H09 17.11

E9.4 4 H06 17.06

E9.4 4 H02 18.75

E9.4 4 H08 16.09

E9.4 4 H03 17.42

E9.4 4 H04 17.16

E9.4 4 H10 14.10

E9.4 4 H11 15.44

E9.4 4 H01 15.89

E10.3 1 H01 11.71

E10.3 1 H02 12.53

E10.3 1 H03 12.74

E10.3 1 H04 10.18

E10.3 1 H05 11.34

E10.3 1 H06 10.56

E10.3 1 H07 11.57

E10.3 1 H08 12.29

E10.3 1 H09 7.67

E10.3 1 H10 10.57

E10.3 1 H11 11.35

E10.3 2 H06 7.50

E10.3 2 H02 13.76

E10.3 2 H09 8.64

E10.3 2 H10 10.89

E10.3 2 H04 9.21

E10.3 2 H07 11.22

E10.3 2 H03 11.74

E10.3 2 H08 11.71

E10.3 2 H01 10.61

E10.3 2 H05 11.30

E10.3 2 H11 10.69

E10.3 3 H09 7.37

E10.3 3 H04 11.56

E10.3 3 H06 10.78

E10.3 3 H11 11.16

E10.3 3 H03 11.90

E10.3 3 H02 11.93

E10.3 3 H07 11.22

E10.3 3 H05 10.93

E10.3 3 H10 9.32

E10.3 3 H08 11.36

E10.3 3 H01 10.58

E10.3 4 H07 11.13

E10.3 4 H05 11.94

E10.3 4 H09 7.78

E10.3 4 H06 9.92

E10.3 4 H02 10.45

E10.3 4 H08 10.21

E10.3 4 H03 12.33

E10.3 4 H04 9.57

E10.3 4 H10 9.34

E10.3 4 H11 10.38

E10.3 4 H01 8.86

E10.4 1 H01 11.48

E10.4 1 H02 12.02

E10.4 1 H03 11.67

E10.4 1 H04 10.19

E10.4 1 H05 10.92

E10.4 1 H06 9.54

E10.4 1 H07 11.97

E10.4 1 H08 12.52

E10.4 1 H09 8.28

E10.4 1 H10 10.04

E10.4 1 H11 11.23

E10.4 2 H06 7.49

E10.4 2 H02 14.30

E10.4 2 H09 10.95

E10.4 2 H10 10.04

E10.4 2 H04 9.78

E10.4 2 H07 11.82

E10.4 2 H03 11.08

E10.4 2 H08 11.95

E10.4 2 H01 10.73

E10.4 2 H05 10.81

E10.4 2 H11 10.11

E10.4 3 H09 8.01

E10.4 3 H04 11.15

E10.4 3 H06 11.64

E10.4 3 H11 11.45

E10.4 3 H03 11.54

E10.4 3 H02 11.68

E10.4 3 H07 11.81

E10.4 3 H05 9.94

E10.4 3 H10 10.09

E10.4 3 H08 11.03

E10.4 3 H01 10.56

E10.4 4 H07 12.06

E10.4 4 H05 10.95

E10.4 4 H09 8.86

E10.4 4 H06 10.33

E10.4 4 H02 10.59

E10.4 4 H08 10.55

E10.4 4 H03 12.75

E10.4 4 H04 9.58

E10.4 4 H10 9.40

E10.4 4 H11 10.52

E10.4 4 H01 9.00
