# Supplementary material for: ProbStab: A probabilistic ML-assisted pipeline for genotype performance, stability, and risk evaluation in multi-environment trials
Source: PLoS One. 2026 Jul 10;21(7):e0352098. doi: 10.1371/journal.pone.0352098 (PMC13354077; doi:10.1371/journal.pone.0352098)
Supplement: S2 File — (DOCX) [file pone.0352098.s012.docx]

environment rep genotype yield

E1.3 1 H01 14.45

E1.3 1 H02 14.16

E1.3 1 H03 15.57

E1.3 1 H04 14.56

E1.3 1 H05 15.23

E1.3 1 H06 13.49

E1.3 1 H07 12.52

E1.3 1 H08 14.52

E1.3 1 H09 11.05

E1.3 1 H10 11.49

E1.3 1 H11 12.97

E1.3 2 H06 13.20

E1.3 2 H02 13.96

E1.3 2 H09 13.74

E1.3 2 H10 15.06

E1.3 2 H04 16.04

E1.3 2 H07 12.72

E1.3 2 H03 13.03

E1.3 2 H08 17.67

E1.3 2 H01 15.23

E1.3 2 H05 13.09

E1.3 2 H11 14.46

E1.3 3 H09 13.41

E1.3 3 H04 13.75

E1.3 3 H06 14.93

E1.3 3 H11 15.03

E1.3 3 H03 15.54

E1.3 3 H02 15.19

E1.3 3 H07 15.90

E1.3 3 H05 14.06

E1.3 3 H10 15.86

E1.3 3 H08 15.46

E1.3 3 H01 17.26

E1.3 4 H07 16.42

E1.3 4 H05 16.87

E1.3 4 H09 15.73

E1.3 4 H06 16.64

E1.3 4 H02 15.21

E1.3 4 H08 16.95

E1.3 4 H03 12.01

E1.3 4 H04 16.35

E1.3 4 H10 15.54

E1.3 4 H11 15.17

E1.3 4 H01 15.29

E1.4 1 H01 15.83

E1.4 1 H02 17.38

E1.4 1 H03 21.87

E1.4 1 H04 16.81

E1.4 1 H05 21.20

E1.4 1 H06 16.06

E1.4 1 H07 19.59

E1.4 1 H08 13.56

E1.4 1 H09 13.45

E1.4 1 H10 15.09

E1.4 1 H11 13.35

E1.4 2 H06 14.80

E1.4 2 H02 15.94

E1.4 2 H09 17.86

E1.4 2 H10 15.44

E1.4 2 H04 14.70

E1.4 2 H07 18.39

E1.4 2 H03 19.13

E1.4 2 H08 13.46

E1.4 2 H01 14.76

E1.4 2 H05 19.23

E1.4 2 H11 16.51

E1.4 3 H09 16.73

E1.4 3 H04 16.07

E1.4 3 H06 12.79

E1.4 3 H11 17.88

E1.4 3 H03 21.22

E1.4 3 H02 16.47

E1.4 3 H07 14.56

E1.4 3 H05 15.53

E1.4 3 H10 13.20

E1.4 3 H08 16.45

E1.4 3 H01 12.48

E1.4 4 H07 17.37

E1.4 4 H05 20.14

E1.4 4 H09 17.77

E1.4 4 H06 17.35

E1.4 4 H02 17.50

E1.4 4 H08 17.06

E1.4 4 H03 16.51

E1.4 4 H04 12.47

E1.4 4 H10 12.28

E1.4 4 H11 16.28

E1.4 4 H01 13.65

E2.3 1 H01 10.97

E2.3 1 H02 14.38

E2.3 1 H03 15.98

E2.3 1 H04 11.61

E2.3 1 H05 6.45

E2.3 1 H06 5.33

E2.3 1 H07 9.36

E2.3 1 H08 7.19

E2.3 1 H09 7.66

E2.3 1 H10 6.61

E2.3 1 H11 6.80

E2.3 2 H06 10.00

E2.3 2 H02 16.11

E2.3 2 H09 7.88

E2.3 2 H10 8.79

E2.3 2 H04 10.87

E2.3 2 H07 13.74

E2.3 2 H03 11.70

E2.3 2 H08 6.36

E2.3 2 H01 7.41

E2.3 2 H05 5.05

E2.3 2 H11 6.07

E2.3 3 H09 7.01

E2.3 3 H04 13.84

E2.3 3 H06 11.82

E2.3 3 H11 10.58

E2.3 3 H03 10.59

E2.3 3 H02 12.08

E2.3 3 H07 12.39

E2.3 3 H05 7.36

E2.3 3 H10 5.90

E2.3 3 H08 9.97

E2.3 3 H01 9.15

E2.3 4 H07 10.02

E2.3 4 H05 10.01

E2.3 4 H09 6.45

E2.3 4 H06 11.61

E2.3 4 H02 12.24

E2.3 4 H08 9.33

E2.3 4 H03 10.86

E2.3 4 H04 12.87

E2.3 4 H10 6.06

E2.3 4 H11 8.90

E2.3 4 H01 10.21

E2.4 1 H01 13.04

E2.4 1 H02 10.79

E2.4 1 H03 13.05

E2.4 1 H04 11.61

E2.4 1 H05 10.92

E2.4 1 H06 11.56

E2.4 1 H07 14.28

E2.4 1 H08 10.81

E2.4 1 H09 7.24

E2.4 1 H10 8.50

E2.4 1 H11 10.77

E2.4 2 H06 12.99

E2.4 2 H02 14.00

E2.4 2 H09 9.52

E2.4 2 H10 11.20

E2.4 2 H04 13.53

E2.4 2 H07 14.18

E2.4 2 H03 12.02

E2.4 2 H08 13.68

E2.4 2 H01 14.34

E2.4 2 H05 15.08

E2.4 2 H11 13.62

E2.4 3 H09 10.63

E2.4 3 H04 14.96

E2.4 3 H06 12.36

E2.4 3 H11 13.72

E2.4 3 H03 13.99

E2.4 3 H02 14.13

E2.4 3 H07 14.80

E2.4 3 H05 15.67

E2.4 3 H10 9.52

E2.4 3 H08 13.50

E2.4 3 H01 12.80

E2.4 4 H07 13.18

E2.4 4 H05 13.41

E2.4 4 H09 11.20

E2.4 4 H06 14.23

E2.4 4 H02 15.03

E2.4 4 H08 14.89

E2.4 4 H03 14.51

E2.4 4 H04 13.38

E2.4 4 H10 11.31

E2.4 4 H11 13.08

E2.4 4 H01 13.30

E4.3 1 H01 17.98

E4.3 1 H02 17.60

E4.3 1 H03 20.94

E4.3 1 H04 17.34

E4.3 1 H05 15.79

E4.3 1 H06 17.48

E4.3 1 H07 16.96

E4.3 1 H08 13.78

E4.3 1 H09 16.88

E4.3 1 H10 11.57

E4.3 1 H11 13.83

E4.3 2 H06 19.42

E4.3 2 H02 19.54

E4.3 2 H09 17.55

E4.3 2 H10 17.37

E4.3 2 H04 16.16

E4.3 2 H07 18.89

E4.3 2 H03 19.13

E4.3 2 H08 16.32

E4.3 2 H01 18.39

E4.3 2 H05 14.88

E4.3 2 H11 14.85

E4.3 3 H09 17.01

E4.3 3 H04 16.01

E4.3 3 H06 18.41

E4.3 3 H11 15.80

E4.3 3 H03 17.47

E4.3 3 H02 18.45

E4.3 3 H07 17.22

E4.3 3 H05 16.53

E4.3 3 H10 14.43

E4.3 3 H08 13.86

E4.3 3 H01 16.37

E4.3 4 H07 17.90

E4.3 4 H05 18.65

E4.3 4 H09 19.65

E4.3 4 H06 17.61

E4.3 4 H02 16.34

E4.3 4 H08 15.93

E4.3 4 H03 17.96

E4.3 4 H04 15.32

E4.3 4 H10 12.84

E4.3 4 H11 14.42

E4.3 4 H01 16.64

E4.4 1 H01 19.38

E4.4 1 H02 17.45

E4.4 1 H03 19.72

E4.4 1 H04 15.76

E4.4 1 H05 16.02

E4.4 1 H06 16.19

E4.4 1 H07 11.81

E4.4 1 H08 17.91

E4.4 1 H09 15.41

E4.4 1 H10 12.22

E4.4 1 H11 13.49

E4.4 2 H06 14.79

E4.4 2 H02 18.80

E4.4 2 H09 16.03

E4.4 2 H10 15.22

E4.4 2 H04 18.37

E4.4 2 H07 14.02

E4.4 2 H03 19.29

E4.4 2 H08 19.38

E4.4 2 H01 20.28

E4.4 2 H05 15.87

E4.4 2 H11 15.05

E4.4 3 H09 12.59

E4.4 3 H04 16.26

E4.4 3 H06 18.92

E4.4 3 H11 18.11

E4.4 3 H03 17.93

E4.4 3 H02 15.38

E4.4 3 H07 11.88

E4.4 3 H05 16.74

E4.4 3 H10 11.84

E4.4 3 H08 15.87

E4.4 3 H01 17.63

E4.4 4 H07 14.29

E4.4 4 H05 17.41

E4.4 4 H09 18.75

E4.4 4 H06 15.44

E4.4 4 H02 17.98

E4.4 4 H08 18.05

E4.4 4 H03 17.60

E4.4 4 H04 18.28

E4.4 4 H10 14.34

E4.4 4 H11 15.25

E4.4 4 H01 13.92

E5.3 1 H01 12.79

E5.3 1 H02 13.01

E5.3 1 H03 12.16

E5.3 1 H04 11.59

E5.3 1 H05 10.09

E5.3 1 H06 8.26

E5.3 1 H07 9.00

E5.3 1 H08 11.01

E5.3 1 H09 8.13

E5.3 1 H10 10.38

E5.3 1 H11 14.92

E5.3 2 H06 8.28

E5.3 2 H02 12.57

E5.3 2 H09 9.94

E5.3 2 H10 10.27

E5.3 2 H04 8.68

E5.3 2 H07 8.55

E5.3 2 H03 10.89

E5.3 2 H08 1.05

E5.3 2 H01 12.10

E5.3 2 H05 7.63

E5.3 2 H11 12.34

E5.3 3 H09 10.34

E5.3 3 H04 8.99

E5.3 3 H06 8.27

E5.3 3 H11 14.62

E5.3 3 H03 11.44

E5.3 3 H02 13.12

E5.3 3 H07 10.99

E5.3 3 H05 8.24

E5.3 3 H10 9.93

E5.3 3 H08 12.33

E5.3 3 H01 13.13

E5.3 4 H07 10.01

E5.3 4 H05 8.38

E5.3 4 H09 8.98

E5.3 4 H06 6.96

E5.3 4 H02 11.83

E5.3 4 H08 12.00

E5.3 4 H03 12.48

E5.3 4 H04 9.67

E5.3 4 H10 10.13

E5.3 4 H11 11.60

E5.3 4 H01 13.36

E5.4 1 H01 8.85

E5.4 1 H02 11.10

E5.4 1 H03 13.35

E5.4 1 H04 11.57

E5.4 1 H05 11.22

E5.4 1 H06 11.96

E5.4 1 H07 11.27

E5.4 1 H08 11.08

E5.4 1 H09 8.69

E5.4 1 H10 8.41

E5.4 1 H11 8.69

E5.4 2 H06 10.13

E5.4 2 H02 13.48

E5.4 2 H09 9.84

E5.4 2 H10 12.25

E5.4 2 H04 14.31

E5.4 2 H07 10.66

E5.4 2 H03 14.28

E5.4 2 H08 12.72

E5.4 2 H01 13.38

E5.4 2 H05 9.30

E5.4 2 H11 11.75

E5.4 3 H09 10.24

E5.4 3 H04 14.13

E5.4 3 H06 9.24

E5.4 3 H11 13.40

E5.4 3 H03 14.72

E5.4 3 H02 15.01

E5.4 3 H07 12.12

E5.4 3 H05 14.10

E5.4 3 H10 13.54

E5.4 3 H08 12.55

E5.4 3 H01 15.38

E5.4 4 H07 8.28

E5.4 4 H05 9.19

E5.4 4 H09 10.74

E5.4 4 H06 13.54

E5.4 4 H02 13.21

E5.4 4 H08 13.69

E5.4 4 H03 14.92

E5.4 4 H04 13.64

E5.4 4 H10 9.26

E5.4 4 H11 11.27

E5.4 4 H01 12.94

E3.3 1 H01 7.41

E3.3 1 H02 10.15

E3.3 1 H03 11.70

E3.3 1 H04 11.60

E3.3 1 H05 9.19

E3.3 1 H06 11.48

E3.3 1 H07 12.73

E3.3 1 H08 5.27

E3.3 1 H09 7.07

E3.3 1 H10 7.18

E3.3 1 H11 12.98

E3.3 2 H06 6.97

E3.3 2 H02 12.90

E3.3 2 H09 10.54

E3.3 2 H10 8.24

E3.3 2 H04 12.03

E3.3 2 H07 14.77

E3.3 2 H03 10.92

E3.3 2 H08 8.55

E3.3 2 H01 9.82

E3.3 2 H05 9.26

E3.3 2 H11 17.83

E3.3 3 H09 8.89

E3.3 3 H04 16.89

E3.3 3 H06 12.07

E3.3 3 H11 15.35

E3.3 3 H03 12.91

E3.3 3 H02 13.71

E3.3 3 H07 15.64

E3.3 3 H05 9.47

E3.3 3 H10 7.19

E3.3 3 H08 11.47

E3.3 3 H01 12.09

E3.3 4 H07 15.77

E3.3 4 H05 10.61

E3.3 4 H09 9.86

E3.3 4 H06 10.32

E3.3 4 H02 12.71

E3.3 4 H08 8.16

E3.3 4 H03 12.21

E3.3 4 H04 9.75

E3.3 4 H10 6.83

E3.3 4 H11 14.23

E3.3 4 H01 15.89

E3.4 1 H01 13.58

E3.4 1 H02 19.57

E3.4 1 H03 26.44

E3.4 1 H04 16.74

E3.4 1 H05 13.09

E3.4 1 H06 14.48

E3.4 1 H07 16.32

E3.4 1 H08 16.15

E3.4 1 H09 11.64

E3.4 1 H10 18.16

E3.4 1 H11 18.75

E3.4 2 H06 19.07

E3.4 2 H02 25.17

E3.4 2 H09 17.05

E3.4 2 H10 14.45

E3.4 2 H04 18.82

E3.4 2 H07 17.03

E3.4 2 H03 17.79

E3.4 2 H08 19.58

E3.4 2 H01 13.45

E3.4 2 H05 16.54

E3.4 2 H11 21.36

E3.4 3 H09 22.12

E3.4 3 H04 15.61

E3.4 3 H06 15.37

E3.4 3 H11 18.76

E3.4 3 H03 23.33

E3.4 3 H02 15.77

E3.4 3 H07 24.87

E3.4 3 H05 13.50

E3.4 3 H10 18.23

E3.4 3 H08 11.96

E3.4 3 H01 26.41

E3.4 4 H07 22.88

E3.4 4 H05 17.91

E3.4 4 H09 16.60

E3.4 4 H06 18.03

E3.4 4 H02 25.19

E3.4 4 H08 24.57

E3.4 4 H03 25.09

E3.4 4 H04 21.80

E3.4 4 H10 18.96

E3.4 4 H11 23.20

E3.4 4 H01 17.27

E6.3 1 H01 13.32

E6.3 1 H02 10.86

E6.3 1 H03 12.00

E6.3 1 H04 10.85

E6.3 1 H05 14.37

E6.3 1 H06 11.64

E6.3 1 H07 12.89

E6.3 1 H08 9.07

E6.3 1 H09 15.77

E6.3 1 H10 12.51

E6.3 1 H11 9.85

E6.3 2 H06 8.11

E6.3 2 H02 8.96

E6.3 2 H09 13.29

E6.3 2 H10 11.79

E6.3 2 H04 10.20

E6.3 2 H07 11.01

E6.3 2 H03 12.08

E6.3 2 H08 10.39

E6.3 2 H01 9.04

E6.3 2 H05 9.51

E6.3 2 H11 11.22

E6.3 3 H09 12.35

E6.3 3 H04 8.90

E6.3 3 H06 11.78

E6.3 3 H11 8.60

E6.3 3 H03 11.35

E6.3 3 H02 9.34

E6.3 3 H07 9.63

E6.3 3 H05 7.89

E6.3 3 H10 11.01

E6.3 3 H08 9.98

E6.3 3 H01 9.03

E6.3 4 H07 9.21

E6.3 4 H05 11.67

E6.3 4 H09 9.75

E6.3 4 H06 8.96

E6.3 4 H02 14.73

E6.3 4 H08 7.71

E6.3 4 H03 12.25

E6.3 4 H04 8.82

E6.3 4 H10 9.34

E6.3 4 H11 9.47

E6.3 4 H01 9.76

E6.4 1 H01 15.99

E6.4 1 H02 14.24

E6.4 1 H03 15.63

E6.4 1 H04 11.49

E6.4 1 H05 13.62

E6.4 1 H06 7.97

E6.4 1 H07 16.78

E6.4 1 H08 12.62

E6.4 1 H09 14.31

E6.4 1 H10 10.73

E6.4 1 H11 8.64

E6.4 2 H06 13.72

E6.4 2 H02 13.15

E6.4 2 H09 15.40

E6.4 2 H10 12.93

E6.4 2 H04 15.25

E6.4 2 H07 16.07

E6.4 2 H03 15.17

E6.4 2 H08 12.66

E6.4 2 H01 12.03

E6.4 2 H05 9.88

E6.4 2 H11 7.57

E6.4 3 H09 12.72

E6.4 3 H04 11.50

E6.4 3 H06 14.81

E6.4 3 H11 11.44

E6.4 3 H03 16.39

E6.4 3 H02 10.95

E6.4 3 H07 13.29

E6.4 3 H05 14.79

E6.4 3 H10 13.03

E6.4 3 H08 7.57

E6.4 3 H01 7.45

E6.4 4 H07 13.75

E6.4 4 H05 10.44

E6.4 4 H09 11.84

E6.4 4 H06 10.79

E6.4 4 H02 15.53

E6.4 4 H08 12.83

E6.4 4 H03 15.33

E6.4 4 H04 13.52

E6.4 4 H10 11.09

E6.4 4 H11 11.35

E6.4 4 H01 6.16

E7.3 1 H01 14.09

E7.3 1 H02 18.74

E7.3 1 H03 15.84

E7.3 1 H04 16.58

E7.3 1 H05 14.83

E7.3 1 H06 15.18

E7.3 1 H07 13.71

E7.3 1 H08 13.57

E7.3 1 H09 17.64

E7.3 1 H10 13.36

E7.3 1 H11 13.40

E7.3 2 H06 15.12

E7.3 2 H02 18.57

E7.3 2 H09 17.00

E7.3 2 H10 15.18

E7.3 2 H04 16.15

E7.3 2 H07 14.39

E7.3 2 H03 15.83

E7.3 2 H08 14.00

E7.3 2 H01 15.49

E7.3 2 H05 15.99

E7.3 2 H11 14.88

E7.3 3 H09 16.93

E7.3 3 H04 18.11

E7.3 3 H06 16.95

E7.3 3 H11 15.16

E7.3 3 H03 17.32

E7.3 3 H02 20.18

E7.3 3 H07 16.11

E7.3 3 H05 17.58

E7.3 3 H10 15.49

E7.3 3 H08 15.16

E7.3 3 H01 15.76

E7.3 4 H07 15.49

E7.3 4 H05 15.89

E7.3 4 H09 17.26

E7.3 4 H06 17.33

E7.3 4 H02 20.26

E7.3 4 H08 14.62

E7.3 4 H03 18.77

E7.3 4 H04 18.61

E7.3 4 H10 14.82

E7.3 4 H11 14.94

E7.3 4 H01 13.92

E7.4 1 H01 14.95

E7.4 1 H02 17.19

E7.4 1 H03 17.69

E7.4 1 H04 18.51

E7.4 1 H05 17.41

E7.4 1 H06 18.46

E7.4 1 H07 18.07

E7.4 1 H08 19.03

E7.4 1 H09 16.09

E7.4 1 H10 13.18

E7.4 1 H11 15.06

E7.4 2 H06 16.49

E7.4 2 H02 17.92

E7.4 2 H09 15.38

E7.4 2 H10 16.24

E7.4 2 H04 18.54

E7.4 2 H07 18.63

E7.4 2 H03 19.29

E7.4 2 H08 18.53

E7.4 2 H01 16.78

E7.4 2 H05 18.54

E7.4 2 H11 15.68

E7.4 3 H09 15.37

E7.4 3 H04 17.01

E7.4 3 H06 15.02

E7.4 3 H11 13.43

E7.4 3 H03 14.70

E7.4 3 H02 12.97

E7.4 3 H07 15.81

E7.4 3 H05 18.47

E7.4 3 H10 13.78

E7.4 3 H08 17.82

E7.4 3 H01 15.29

E7.4 4 H07 13.64

E7.4 4 H05 15.37

E7.4 4 H09 15.96

E7.4 4 H06 15.53

E7.4 4 H02 15.45

E7.4 4 H08 15.75

E7.4 4 H03 15.11

E7.4 4 H04 16.77

E7.4 4 H10 14.46

E7.4 4 H11 13.20

E7.4 4 H01 14.65

E8.3 1 H01 5.23

E8.3 1 H02 6.69

E8.3 1 H03 7.27

E8.3 1 H04 7.02

E8.3 1 H05 4.66

E8.3 1 H06 4.69

E8.3 1 H07 7.00

E8.3 1 H08 6.07

E8.3 1 H09 4.07

E8.3 1 H10 5.69

E8.3 1 H11 6.69

E8.3 2 H06 5.62

E8.3 2 H02 6.24

E8.3 2 H09 5.34

E8.3 2 H10 4.51

E8.3 2 H04 5.16

E8.3 2 H07 6.12

E8.3 2 H03 6.17

E8.3 2 H08 5.55

E8.3 2 H01 6.76

E8.3 2 H05 5.31

E8.3 2 H11 5.20

E8.3 3 H09 4.82

E8.3 3 H04 7.36

E8.3 3 H06 5.94

E8.3 3 H11 5.87

E8.3 3 H03 5.83

E8.3 3 H02 5.75

E8.3 3 H07 6.62

E8.3 3 H05 5.43

E8.3 3 H10 5.74

E8.3 3 H08 5.02

E8.3 3 H01 6.11

E8.3 4 H07 6.45

E8.3 4 H05 5.08

E8.3 4 H09 4.74

E8.3 4 H06 5.18

E8.3 4 H02 4.88

E8.3 4 H08 4.80

E8.3 4 H03 6.85

E8.3 4 H04 8.55

E8.3 4 H10 6.42

E8.3 4 H11 6.39

E8.3 4 H01 6.46

E8.4 1 H01 5.76

E8.4 1 H02 5.92

E8.4 1 H03 6.22

E8.4 1 H04 7.87

E8.4 1 H05 4.84

E8.4 1 H06 4.68

E8.4 1 H07 7.28

E8.4 1 H08 5.61

E8.4 1 H09 4.56

E8.4 1 H10 4.54

E8.4 1 H11 4.51

E8.4 2 H06 2.49

E8.4 2 H02 4.65

E8.4 2 H09 3.08

E8.4 2 H10 4.05

E8.4 2 H04 15.58

E8.4 2 H07 5.88

E8.4 2 H03 6.89

E8.4 2 H08 5.53

E8.4 2 H01 7.34

E8.4 2 H05 4.32

E8.4 2 H11 4.96

E8.4 3 H09 3.84

E8.4 3 H04 4.37

E8.4 3 H06 4.43

E8.4 3 H11 4.88

E8.4 3 H03 3.65

E8.4 3 H02 4.91

E8.4 3 H07 6.43

E8.4 3 H05 6.22

E8.4 3 H10 5.47

E8.4 3 H08 6.94

E8.4 3 H01 5.83

E8.4 4 H07 6.80

E8.4 4 H05 3.79

E8.4 4 H09 4.39

E8.4 4 H06 6.09

E8.4 4 H02 4.97

E8.4 4 H08 5.82

E8.4 4 H03 6.59

E8.4 4 H04 6.39

E8.4 4 H10 3.39

E8.4 4 H11 5.19

E8.4 4 H01 7.67

E9.3 1 H01 9.22

E9.3 1 H02 9.48

E9.3 1 H03 9.79

E9.3 1 H04 8.07

E9.3 1 H05 10.10

E9.3 1 H06 8.26

E9.3 1 H07 10.54

E9.3 1 H08 8.78

E9.3 1 H09 10.50

E9.3 1 H10 10.11

E9.3 1 H11 8.57

E9.3 2 H06 8.68

E9.3 2 H02 8.86

E9.3 2 H09 10.36

E9.3 2 H10 9.32

E9.3 2 H04 6.28

E9.3 2 H07 10.85

E9.3 2 H03 7.97

E9.3 2 H08 6.61

E9.3 2 H01 6.83

E9.3 2 H05 9.05

E9.3 2 H11 10.43

E9.3 3 H09 13.91

E9.3 3 H04 10.24

E9.3 3 H06 10.22

E9.3 3 H11 12.72

E9.3 3 H03 14.79

E9.3 3 H02 12.08

E9.3 3 H07 13.85

E9.3 3 H05 12.51

E9.3 3 H10 9.72

E9.3 3 H08 11.60

E9.3 3 H01 11.85

E9.3 4 H07 11.90

E9.3 4 H05 12.17

E9.3 4 H09 12.65

E9.3 4 H06 11.53

E9.3 4 H02 12.83

E9.3 4 H08 12.99

E9.3 4 H03 12.49

E9.3 4 H04 11.15

E9.3 4 H10 8.51

E9.3 4 H11 10.03

E9.3 4 H01 13.95

E9.4 1 H01 16.45

E9.4 1 H02 16.22

E9.4 1 H03 18.72

E9.4 1 H04 17.13

E9.4 1 H05 19.22

E9.4 1 H06 15.90

E9.4 1 H07 13.83

E9.4 1 H08 16.10

E9.4 1 H09 15.62

E9.4 1 H10 14.92

E9.4 1 H11 14.74

E9.4 2 H06 12.68

E9.4 2 H02 16.02

E9.4 2 H09 16.66

E9.4 2 H10 15.01

E9.4 2 H04 14.85

E9.4 2 H07 14.86

E9.4 2 H03 16.44

E9.4 2 H08 17.53

E9.4 2 H01 17.96

E9.4 2 H05 14.88

E9.4 2 H11 15.38

E9.4 3 H09 14.43

E9.4 3 H04 16.47

E9.4 3 H06 15.21

E9.4 3 H11 14.73

E9.4 3 H03 16.93

E9.4 3 H02 16.84

E9.4 3 H07 15.51

E9.4 3 H05 15.04

E9.4 3 H10 16.75

E9.4 3 H08 13.10

E9.4 3 H01 14.79

E9.4 4 H07 20.39

E9.4 4 H05 13.42

E9.4 4 H09 17.11

E9.4 4 H06 17.06

E9.4 4 H02 18.75

E9.4 4 H08 16.09

E9.4 4 H03 17.42

E9.4 4 H04 17.16

E9.4 4 H10 14.10

E9.4 4 H11 15.44

E9.4 4 H01 15.89

E10.3 1 H01 11.71

E10.3 1 H02 12.53

E10.3 1 H03 12.74

E10.3 1 H04 10.18

E10.3 1 H05 11.34

E10.3 1 H06 10.56

E10.3 1 H07 11.57

E10.3 1 H08 12.29

E10.3 1 H09 7.67

E10.3 1 H10 10.57

E10.3 1 H11 11.35

E10.3 2 H06 7.50

E10.3 2 H02 13.76

E10.3 2 H09 8.64

E10.3 2 H10 10.89

E10.3 2 H04 9.21

E10.3 2 H07 11.22

E10.3 2 H03 11.74

E10.3 2 H08 11.71

E10.3 2 H01 10.61

E10.3 2 H05 11.30

E10.3 2 H11 10.69

E10.3 3 H09 7.37

E10.3 3 H04 11.56

E10.3 3 H06 10.78

E10.3 3 H11 11.16

E10.3 3 H03 11.90

E10.3 3 H02 11.93

E10.3 3 H07 11.22

E10.3 3 H05 10.93

E10.3 3 H10 9.32

E10.3 3 H08 11.36

E10.3 3 H01 10.58

E10.3 4 H07 11.13

E10.3 4 H05 11.94

E10.3 4 H09 7.78

E10.3 4 H06 9.92

E10.3 4 H02 10.45

E10.3 4 H08 10.21

E10.3 4 H03 12.33

E10.3 4 H04 9.57

E10.3 4 H10 9.34

E10.3 4 H11 10.38

E10.3 4 H01 8.86

E10.4 1 H01 11.48

E10.4 1 H02 12.02

E10.4 1 H03 11.67

E10.4 1 H04 10.19

E10.4 1 H05 10.92

E10.4 1 H06 9.54

E10.4 1 H07 11.97

E10.4 1 H08 12.52

E10.4 1 H09 8.28

E10.4 1 H10 10.04

E10.4 1 H11 11.23

E10.4 2 H06 7.49

E10.4 2 H02 14.30

E10.4 2 H09 10.95

E10.4 2 H10 10.04

E10.4 2 H04 9.78

E10.4 2 H07 11.82

E10.4 2 H03 11.08

E10.4 2 H08 11.95

E10.4 2 H01 10.73

E10.4 2 H05 10.81

E10.4 2 H11 10.11

E10.4 3 H09 8.01

E10.4 3 H04 11.15

E10.4 3 H06 11.64

E10.4 3 H11 11.45

E10.4 3 H03 11.54

E10.4 3 H02 11.68

E10.4 3 H07 11.81

E10.4 3 H05 9.94

E10.4 3 H10 10.09

E10.4 3 H08 11.03

E10.4 3 H01 10.56

E10.4 4 H07 12.06

E10.4 4 H05 10.95

E10.4 4 H09 8.86

E10.4 4 H06 10.33

E10.4 4 H02 10.59

E10.4 4 H08 10.55

E10.4 4 H03 12.75

E10.4 4 H04 9.58

E10.4 4 H10 9.40

E10.4 4 H11 10.52

E10.4 4 H01 9.00
